# The role of uncertainty and negative feedback loops in the evolution of induced immune defenses

**DOI:** 10.1093/g3journal/jkae182

**Published:** 2024-08-06

**Authors:** Danial Asgari, Alexander J Stewart, Richard P Meisel

**Affiliations:** Department of Biology and Biochemistry, University of Houston, Houston, TX 77204, USA; School of Mathematics and Statistics, University of St Andrews, St Andrews KY16 9AJ, UK; Department of Biology and Biochemistry, University of Houston, Houston, TX 77204, USA

**Keywords:** immunity, negative regulation, bacterial infection, constitutive defense

## Abstract

Organisms use constitutive or induced defenses against pathogens and other external threats. Constitutive defenses are constantly on, whereas induced defenses are activated when needed. Each of these strategies has costs and benefits, which can affect the type of defense that evolves in response to pathogens. In addition, induced defenses are usually regulated by multiple negative feedback mechanisms that prevent overactivation of the immune response. However, it is unclear how negative feedback affects the costs, benefits, and evolution of induced responses. To address this gap, we developed a mechanistic model of the well-characterized *Drosophila melanogaster* immune signaling network that includes 3 separate mechanisms of negative feedback as a representative of the widespread phenomenon of multilevel regulation of induced responses. We show that, under stochastic fly–bacteria encounters, an induced defense is favored when bacterial encounters are rare or uncertain, but in ways that depend on the bacterial proliferation rate. Our model also predicts that the specific negative regulators that optimize the induced response depend on the bacterial proliferation rate, linking negative feedback mechanisms to the factors that favor induction.

## Introduction

Organisms defend themselves against pathogens, parasites, and other threats using induced and constitutive defenses. Induced responses are produced when an organism is attacked, whereas constitutive defenses are always on. Induced and constitutive defenses each have advantages and limitations (i.e. benefits and costs). For example, constitutive defenses may require costly resources because they are always on or have direct deleterious (immunopathological) effects on host tissues (Aggarwal and Silverman 2008; Li *et al*. 2020). Induced strategies may be more cost-effective because defensive molecules are only produced when needed (Karban 2020). However, this induced strategy can create a costly delay in response (Karban and Myers 1989). If the cost of defense is low or delay in response is too costly, a constitutive strategy might therefore be favored (Bixenmann *et al*. 2016).

Mathematical models have revealed how the predictability and certainty of attacks affect the evolution of induced and constitutive defenses. For example, Clark and Harvell (1992) showed that an induced defense is favored when attacks by predators are unpredictable, and Hamilton *et al*. (2008) found that uncertainty about the parasite proliferation rate favors induction. In contrast, increasing the probability of attacks (Shudo and Iwasa 2001; Kamiya *et al*. 2016) and certainty of an attack (Adler and Karban 1994) favor a constitutive defense.

Negative regulation is a key component of induced defenses, which prevents overstimulation of the host response (Luecke *et al*. 2021). Immune signaling pathways across many taxa are modulated by multilevel negative regulation across different steps of signaling. For example, some negative regulators function upstream of signaling pathways and close to immune receptors to reduce input into the signaling pathway, such as A20 in the NF-κB signaling pathway of vertebrates (Shembade and Harhaj 2012). In insects, upon immune signaling, Pirk serves the same function by reducing the number of immune receptors on the cell surface and shutting down signaling before its propagation (Kleino *et al*. 2008). On the other hand, IκBα in vertebrates and the repressosome complex in insects function inside the nucleus to reduce the transcriptional output of the signaling pathway after propagation of the signal (Kim *et al*. 2007; Kamata *et al*. 2010). Despite our mechanistic knowledge of these negative regulators, we do not understand the implication of multilevel regulation of signaling on the evolution of induced responses.

**Fig. 1.**
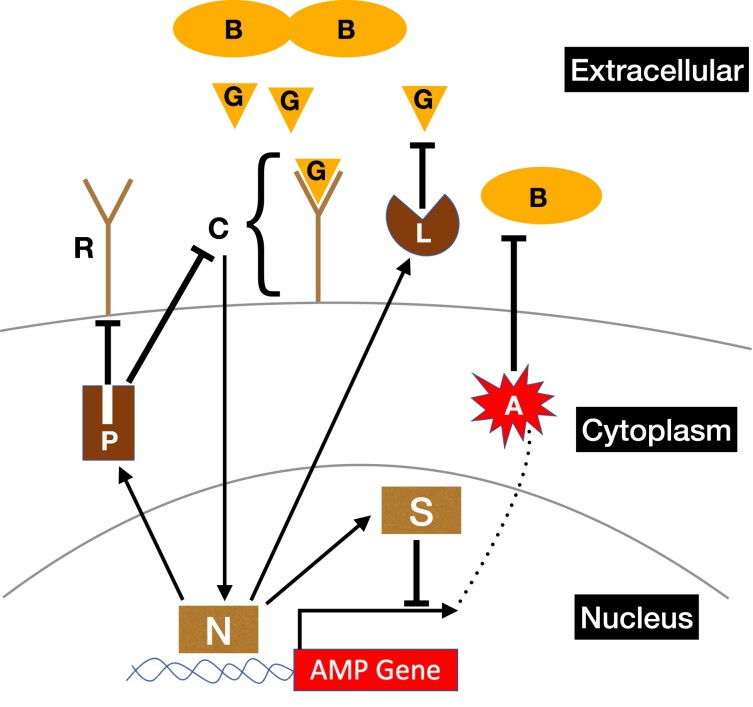
Diagram showing the Imd signaling pathway for induction of antimicrobial peptides. The letters in the diagram correspond to the variables in the system of ordinary differential equations of the induced model (Equations (1)–(9)). Peptidoglycans (G) produced by bacteria (B) bind to cell surface receptors (R), forming a receptor complex (C) that initiates an NF-κB signaling pathway to activate the transcription factor Relish (N). Relish promotes the transcription of AMP genes (A), which leads to the production of AMPs (A). Relish also induces the production of Pirk (P), scavengers of peptidoglycans (L), and at least 1 gene encoding a component of the repressosome complex (S). AMPs destroy bacteria, and Pirk reduces available cell surface receptors. Repressosome (S) competes with Relish (N) for binding to the promoter of AMP genes.

To investigate the relationships between conditions that favor the evolution of the induced response and multilevel negative feedbacks, we developed a detailed mathematical model of the *Drosophila melanogaster* immune deficiency (Imd) pathway (Fig. 1). The Imd pathway is a major signaling cascade involved in the *Drosophila* induced immune response to bacterial infection (De Gregorio *et al*. 2002). The Imd pathway, which is activated by diaminopimelic acid (DAP)-type PG in the cell walls of Gram-negative bacteria (Kaneko *et al*. 2005), includes multiple negative feedbacks that prevent overstimulation, including Pirk and the repressosome complex. We modeled Imd signaling because it is well-characterized and shares many similarities with other immune signaling pathways, including and especially the multilevel regulation of immune signaling by negative feedback loops at different stages of signaling.

Our model includes all of the key components of the Imd pathway. First, the Imd pathway is activated when bacterial PG binds to receptor proteins (PGRP-LC) on the surface of *Drosophila* enterocytes (Kaneko *et al*. 2006; Schmidt *et al*. 2008). Next, a signaling complex—consisting of the Imd protein, the adaptor protein dFADD, and the caspase protein DREDD—is recruited to the intracellular domain of the receptor (Georgel *et al*. 2001; Leulier *et al*. 2002). DREDD cleaves Relish, which is an NF-κB transcription factor (Stoven *et al*. 2003), and the N-terminal domain of Relish is translocated into the nucleus where it facilitates the transcription of AMP genes (Stöven *et al*. 2000). Imd signaling concurrently activates negative multiple feedback loops, which protect the host from harmful effects of excessive immune response (Badinloo *et al*. 2018). First, scavengers of PG (PGRP-LB) are expressed in a Relish-dependent manner, which reduce Imd signaling by removing PGs from the gut (De Gregorio *et al*. 2002). Second, Relish also upregulates *pirk*, which reduces the number of available PGRP-LC receptors on the cell surface (Kleino *et al*. 2008; Lhocine *et al*. 2008). Third, following activation of the Imd pathway, 2 transcription factors, Jra and Stat92Ea, form the repressosome complex with a high mobility group protein DSP1. The repressosome competes with Relish over binding to promoters of AMP genes, thereby reducing AMP expression (Kim *et al*. 2007). The production of the transcription factor Jra has been shown to be regulated by Relish, thus acting as a negative feedback loop to attenuate Imd signaling (De Gregorio *et al*. 2002).

We used a system of ordinary differential equations (ODEs) to model the Imd response to bacteria in *D. melanogaster*. Notably, our model includes the 3 aforementioned negative feedback loops involved in the Imd signaling pathway, which goes beyond the detail in previously developed models (Ellner *et al*. 2021). Our model is therefore the first, to our knowledge, to include mechanistic components of negative regulation. We explored how negative regulators modulating the immune response at different steps in the signaling pathway shape the fitness of an induced defense when the host encounters different bacterial populations. We compared the fitness of this model to a simple model of constitutive defense. We did not consider mixed strategies as it has been shown that immune genes can adopt a purely constitutive or a purely induced strategy (Asgari *et al*. 2022). Our models do not capture the evolution of systems across generations because it is not tractable to do so with a model that includes the mechanistic details we have incorporated. Instead, we focus on comparing the performance of induced and constitutive models using many parameters that reflect realistic aspects of host–pathogen interactions. Both our induced and constitutive models include features that capture the bacterial proliferation rate and survival in the gut, which has been shown experimentally to vary across bacteria species (Duneau *et al*. 2017; Pais *et al*. 2018). We considered stochastic encounters of flies with bacteria in a variety of environments, with different bacterial density and patchiness, to determine how bacterial exposure and specific negative feedbacks affect the conditions in which induced or constitutive defenses are favored.

## Materials and methods

### The model

We modeled the Imd pathway using a system of ODEs which capture the dynamics of immune response to bacterial infection (Fig. 1). Equation (1) describes the change in bacterial density (*B*) in the gut of an individual fly.


(1) $$\frac{dB}{dt}=f(t)+k_{0}B_{t}-A_{t}B_{t}$$

Under our model, the density of bacteria entering the gut at time *t* is described by the function f(t). In general, f(t) depends on the local concentration of bacteria encountered by the fly as it moves through its environment. For example, we may consider scenarios in which f(t) is a deterministic, oscillating (sinusoidal) function. We also consider scenarios in which f(t) describes the probability of entering or leaving a patch in an environment with randomly distributed colonies of bacteria. After ingestion, bacteria proliferate inside the gut at rate $k_{0}$, and are killed by AMPs at a rate $A_{t}B_{t}$, where $A_{t}$ is the concentration of AMP at time *t*. In order to contrast the Imd pathway with a constitutive immune response, we modeled constitutive expression by simply assuming that $A_{t}$ is a constant ($A_{t}=A$) in Equation (1). The model for the constitutive defense only contains Equation (1) because signaling proteins are not involved in constitutive defenses.

The concentration of free bacterial PG at time *t*, ($G_{t}$), is described by Equation (2).


(2) $$\frac{dG}{dt}=\;\alpha k_{0}B_{t}-L_{t}G_{t}-R_{t}G_{t}+P_{t}C_{t}+\lambda_{3}C_{t}-\lambda_{1}G_{t}$$

The production of free PG is stimulated by the proliferation of bacteria ($k_{0}B_{t}$) at rate *α*. This ensures that the host responds to proliferating bacteria, as shown by empirical studies (Kurata 2014; Wolf and Underhill 2018). We assume that free PG is scavenged (i.e. removed from the system) by PGRP-LB $\left(L_{t}\right)$ interacting with $G_{t}$ (Zaidman-Rémy *et al*. 2006; Costechareyre *et al*. 2016). Free PG is also lost when it binds to receptors on the cell surface ($R_{t}$). We also assume that PG is released when the PG-receptor complex (produced at a rate $C_{t}$ ) is pulled down by Pirk (produced at a rate $P_{t}$) (Kleino *et al*. 2008). The receptor-PG complex is dissociated at rate $\lambda_{3}$, which produces free PG and free receptors. Finally, PG is degraded at rate $\lambda_{1}$.

Next, we describe the change in concentration of free receptor (*R*) over time via Equation (3).


(3) $$\frac{dR}{dt}=\;R_{0}+\beta_{1}\frac{N_{t}}{N_{t}+Z_{n}}-P_{t}R_{t}-R_{t}G_{t}+\lambda_{3}C_{t}-\lambda_{2}R_{t}$$

Here, receptors are produced at a base rate $R_{0}$. Receptor expression is positively regulated by the NF-κB transcription factor Relish ($N_{t}$) at rate $\beta_{1}$, which is modeled as a Hill function (Liu *et al*. 2020). $Z_{n}$ is the binding energy of Relish to the promoter, which is inversely proportional to the probability of binding. Interactions with Pirk and PG reduce the concentration of free receptors. We considered both separate and identical degradation rates for R,N,L,P,*S*, and *A* (described below). Equations in the main text have equal degradation rates for R,N,L,P,*S*, and *A*$\left(\lambda_{2}\right)$. Following from Equation (3), change in the concentration of receptor-PG complex (*C*) is described by Equation (4).


(4) $$\frac{dC}{dt}=\;R_{t}G_{t}-P_{t}C_{t}-\lambda_{3}C_{t}$$

Equation (5) describes the change in the NF-κB transcription factor Relish, which is activated at rate $\beta_{2}$ following formation of the PG-receptor complex (Stoven *et al*. 2003).


(5) $$\frac{dN}{dt}=\beta_{2}C_{t}-\lambda_{2}N_{t}$$

Equations (6)–(8) describe negative feedback loops mediated by Relish.


(6) $$\frac{dL}{dt}=\beta_{3}\frac{N_{t}}{N_{t}+Z_{n}}-\lambda_{2}L_{t}$$

(7) $$\frac{dP}{dt}=\beta_{4}\frac{N_{t}}{N_{t}+Z_{n}}-\lambda_{2}P_{t}$$

(8) $$\frac{dS}{dt}=\beta_{5}\frac{N_{t}}{N_{t}+Z_{n}}-\lambda_{2}S_{t}$$

PGRP-LB, Pirk, and the repressosome (AP-1 and STAT complex, *S_t_*) are activated by Relish at rates $\beta_{3}$, $\beta_{4}$, and $\beta_{5}$, respectively (Zaidman-Rémy *et al*. 2006; Kim *et al*. 2007; Kleino *et al*. 2008; Lhocine *et al*. 2008). Equation (9) describes AMP expression, which is upregulated by Relish and repressed by the repressosome with a single rate of $\beta_{6}$. The repressosome competes with Relish for binding to promoters of AMPs (Kim *et al*. 2007). The binding energy for the repressosome is $Z_{s}$.


(9) $$\frac{dA}{dt}=\beta_{6}\frac{N_{t}}{N_{t}+Z_{n}+\frac{Z_{n}S_{t}}{Z_{s}}}-\lambda_{2}A_{t}$$

### Simulation of the bacterial environment

**Fig. 2.**
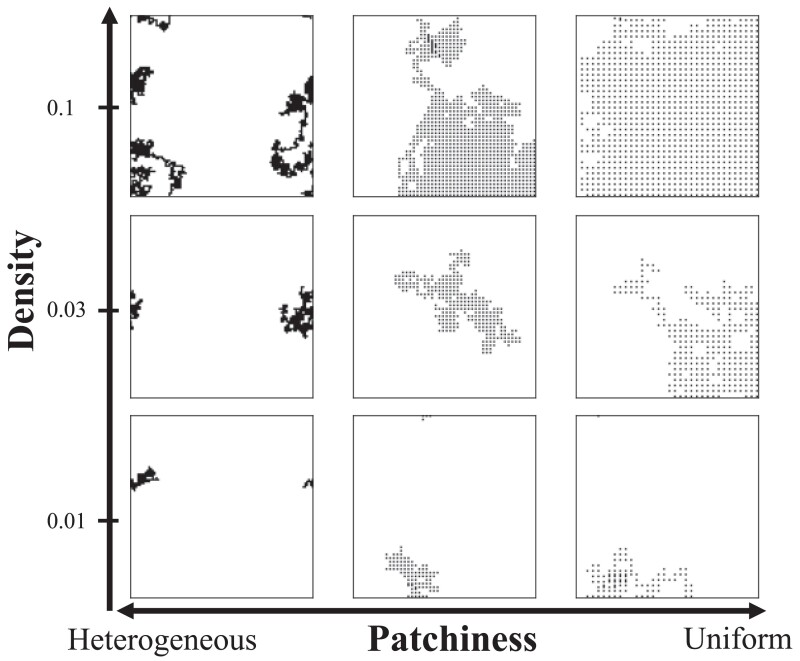
Simulated environments with different numbers (density) and patchiness of bacterial colonies. Each dot represents a bacterial colony on a 100×100 lattice.

In order to capture the effect of stochastic interactions with bacterial colonies on the immune response, we simulated environments with different bacterial distributions. Environments varied in their patchiness, from heterogeneous distributions with clusters of colonies in close proximity (lower *x*-axis values in Fig. 2) to those with more uniform distributions (higher *x*-axis values in Fig. 2). The simulated environments also varied in bacterial density (number of bacteria per area), indicated on the *y*-axis in Fig. 2. For detailed information on the bacterial population simulation algorithm, please refer to the supplemental document.

### Movement of the fly through the environment

We used both a deterministic and stochastic approach to model fly movement through bacterial environments. In the deterministic approach, we considered an oscillating model in which $f(t)=\omega \;\sin(t\;\Phi {)}^{2}$, such that the fly experiences a deterministic oscillating bacterial environment. The frequency of encounter is captured by Φ, and the amount of bacteria is represented by *ω*. For the deterministic oscillating model, we solved the system of ODEs numerically using the scipy python package. Due to the complexity of the ODE system, an analytical solution is not feasible.

In the stochastic method, we assessed the efficacy of constitutive defense and induced immune response by comparing their efficacy over the course of a large number of sojourns through simulated bacterial environments (Fig. 2). To those ends, we simulated the behavior of a fly within a given bacterial environment as a random walk across a lattice seeded with a bacterial distribution as described above (see supplemental methods for details).

Two parameters in our induced model define the interaction between the bacteria that flies encounter and their immune system during their deterministic oscillations and random walks: the rate at which PG is released upon bacterial proliferation (*α*) and the degradation rate of PG ($\lambda_{1}$). We considered 4 values for *α* (0.2,1,2,4) and 2 values for $\lambda_{1}$ (0.01 and 0.05). However, our results focus on $\lambda_{1}$ = 0.01 for reasons described in the *Results*. In both the induced and constitutive models, the efficacy of the immune response also depends on the bacterial proliferation rate ($k_{0}$). We tested both constitutive and induced defenses against 3 different bacterial proliferation rates ($k_{0}$ = 0.1, $k_{0}$ = 0.2, and $k_{0}$ = 0.5).

### Calculating fly fitness

After obtaining a vector containing values at every time point for each variable in the model $\;(B_{t},\;G_{t},\;R_{t},\;C_{t},\;N_{t},\;L_{t},\;P_{t},\;S_{t},\;A_{t})$, we calculated fitness as an exponentially decreasing function of the summed time averaged values of each of the model variables, as shown in Equation (10) for an induced response and in Equation (11) for constitutive defenses.


(10) $$F_{\mathrm{induced}}=e^{-(\underline{B}+\underline{N}+\underline{L}+\underline{P}+\underline{S}+\underline{A})}$$

(11) $$F_{\mathrm{constitutive}}=\;e^{-(\underline{B}+A)}$$

In these calculations (Equations (10) and (11)), $\underline{B}$ is the arithmetic time averaged bacterial concentration in the gut, $\underline{N}$ is the time averaged production of Relish, and so on. This method assumes that fitness declines exponentially with the average bacterial load $\underline{B}$ and with the strength of the immune response due to the cost of production. Here, we assume that the energetic cost associated with production of immune proteins is equal to the costs imposed by the bacteria. Thus, based on Equation (10), an optimum immune response should balance the costs of immunity with those of infection. This assumption is supported by empirical evidence demonstrating the energetic costs of mounting an immune response (Eraud *et al*. 2005; Ardia *et al*. 2012). To test if our results are robust to the choice of fitness function, we also consider a scenario where the fitness of induced defense depends only on AMP production and bacteria, similar to Equation (11). This fitness function evaluates the effects of an immune system in which the energetic costs of protein synthesis are negligible, and instead costs are due to immunopathological effects of AMP production (Badinloo *et al*. 2018).

For each environment with a particular bacterial density and patchiness, we simulated 1,000 fly random walks each consisting of 100,000 steps, and we calculated the arithmetic mean fitness for the constitutive and induced defenses across simulations. The fitness difference (ΔF) between the induced and constitutive immune responses was calculated by subtracting the average fitness of the induced response (${\underline{F}}_{\mathrm{induced}}$) from the average fitness for the constitutive defense (${\underline{F}}_{\mathrm{constitutive}}$) using Equation (12).


(12) $$\Delta F\;={\underline{F}}_{\mathrm{constitutive}}\;-\;\;{\underline{F}}_{\mathrm{induced}}$$

### Parameter optimization

The model for induced response has 11 parameters that describe the attributes of the Imd pathway $(\lambda_{2},\;\lambda_{3},\;R_{0},\;\beta_{1},\;\beta_{2},\;\beta_{3},\;\beta_{4},\;\beta_{5},$$\beta_{6},\;Z_{n},\;Z_{s}$). We identified the parameters that maximized the fitness of the induced response by simulating random walks within the 11-dimensional landscape consisting of the 11 parameters (Supplementary Table 1). Optimization of the induced defense was performed using a sinusoidally oscillating (i.e. deterministic) input because optimization using a stochastic input is too computationally intensive to be tractable. Induced defenses are plastic responses that can evolve when environmental fluctuations are predictable (Scheiner 1993). Therefore, optimizing the induced defense by a sinusoidal function, which simulates predictable fluctuations, is a valid approach for producing effective induced immune defenses. The optimization method is explained in detail in the supplemental document.

To optimize the constitutive defense, the amount of constitutively expressed AMP (*A*) was changed over a range of 0.01–2 (step size = 0.01), and the value of *A* that confers the highest fitness was chosen. The Euler method was used (*h* = 0.01) to solve Equation (1), where $A_{t}$ is a constant ($A_{t}\;=\;A$). Due to the simplicity of the model for constitutive defense, the model was optimized in stochastic environments. The optimum fitness for the constitutive defense was calculated in each combination of bacterial density, patchiness, and proliferation rate and then compared to the fitness of different optimizations of the induced defense (i.e. induced defenses optimized with different values of *Φ*). The induced and constitutive defenses that are tested against each other are optimized using the same bacterial proliferation rate.

### Simulating fluctuation across multiple environments

We simulated fluctuation in density and/or patchiness of bacterial populations. To those ends, we randomly selected *j* environments with different densities and/or patchiness of bacteria, and we assumed that flies encounter those environments with equal probabilities. Sampling was done without replacement in order to maximize fluctuations in the density and/or patchiness of the bacterial population. To estimate the fitness of flies encountering *j* bacterial environments with equal probabilities, we calculated the arithmetic mean of the fitness values across *j* environments (Equation (13)).


(13) $$F_{\mathrm{induction}}=\overset{j}{\underset{i=1}{\sum }}\frac{F_{i}\;}{j}\;\;\;$$

In each of the *j* environments, the fly stochastically encounters bacteria. Simulations were performed for induced defenses that were optimized with different values of *Φ*.

To find the optimum fitness for the constitutive defense when flies inhabit *j* environments with equal probabilities ($j^{-1}$), we calculated the fitness in each environment for constitutive defenses ranging from 0.01 to 2 units of AMP production, (i.e. 0.01≤A≤2), in 0.01 increments. This wide range of values for constitutive AMP production ensures high fitness in environments with different rates of encounters with pathogens. Next, we calculated arithmetic means of fitness values across *j* environments (dashed lines in Supplementary Fig. 1 in File S1) and chose the maximum value (arrows in Supplementary Fig. 1 in File S1). We measured the proportion of times induction outperforms constitutive defense by repeating the process of random selection of environments and calculation of the relative fitness (ΔF) 10,000 times.

We also calculated the relative fitness of the constitutive and induced defenses when the fly inhabits 2 environments with probabilities *q* and 1−q. This differs from the previous analysis, where $q=j^{-1}$. The fitness of the induced response is the weighted average of optimum fitness values across the 2 environments (Equation (14)).


(14) $$F_{\mathrm{induction}}=q\;F_{1}\;+\;(1-q)\;F_{2}$$

We used the following approach to find the optimum value of AMP production (*A*) for the constitutive defense when flies inhabit 2 environments with probabilities *q* and 1−q. First fitness was calculated in each of the 2 environments for constitutive defenses ranging from 0.01 to 2 units of AMP production (0.01≤A≤2). The optimum fitness of constitutive defense is the maximum value (arrows in Supplementary Fig. 2 in File S1) of the weighted averages of fitness values (dashed lines in Supplementary Fig. 2 in File S1). The proportion of times induction outperformed constitutive defense for 10,000 combinations of environments was calculated for each value of *q*, as explained above.

## Results

### Induction is favored when bacteria are at low/intermediate density and have heterogeneous distributions

In order to compare the fitness of our mechanistic model of the induced immune response with constitutive defense, we first found parameter values in our model that maximize fitness for each strategy in a variety of environments. We optimized induced defenses with different frequencies of bacterial exposure (*Φ*) and 4 rates of bacterial PG production (*α* = 0.2, 1, 2, and 4; Supplementary Fig. 3a in File S1). Our results were qualitatively similar across different *α* and *Φ* values (Supplementary Fig. 3b in File S1). For the remainder of the manuscript, we only focus on results for *α* = 2 because it maximizes the performance of induced defenses when flies encounter 2 different bacterial distributions (Supplementary Fig. 3c in File S1). We set the natural degradation rate of PG to $\lambda_{1}$ = 0.01 (for details, refer to the supplemental text and Supplementary Figs. 4 and 5).

We compared the fitness of the induced and constitutive defenses under a sinusoidal mode of encounter with bacteria ($f(t)=\omega \;\sin(t\;\Phi {)}^{2}$). Increasing the frequency of fly–bacteria encounters (*Φ*) reduced the optimal fitness of the induced response, regardless of the bacterial proliferation rate ($k_{0}$) inside the fly (Supplementary Fig. 5 in File S1). When induction was optimized with high frequency of bacterial exposure (*Φ* = 0.1), the induced defense performed poorly when compared to a constitutive defense for most frequencies and amplitudes of exposure (Supplementary Fig. 6 in File S1). When the frequency of encounters with bacteria (Φ) was intermediate, an induced response was favored over constitutive defense for at least some values of amplitude (ω) (Supplementary Fig. 6 in File S1). Our results therefore suggest that induced responses have lower fitness in environments with higher frequencies of fly–bacteria interactions. A limitation of this analysis is that the fly–bacteria encounters are predictable when there is a deterministic sinusoidal input of bacteria, which is not biologically realistic.

**Fig. 3.**
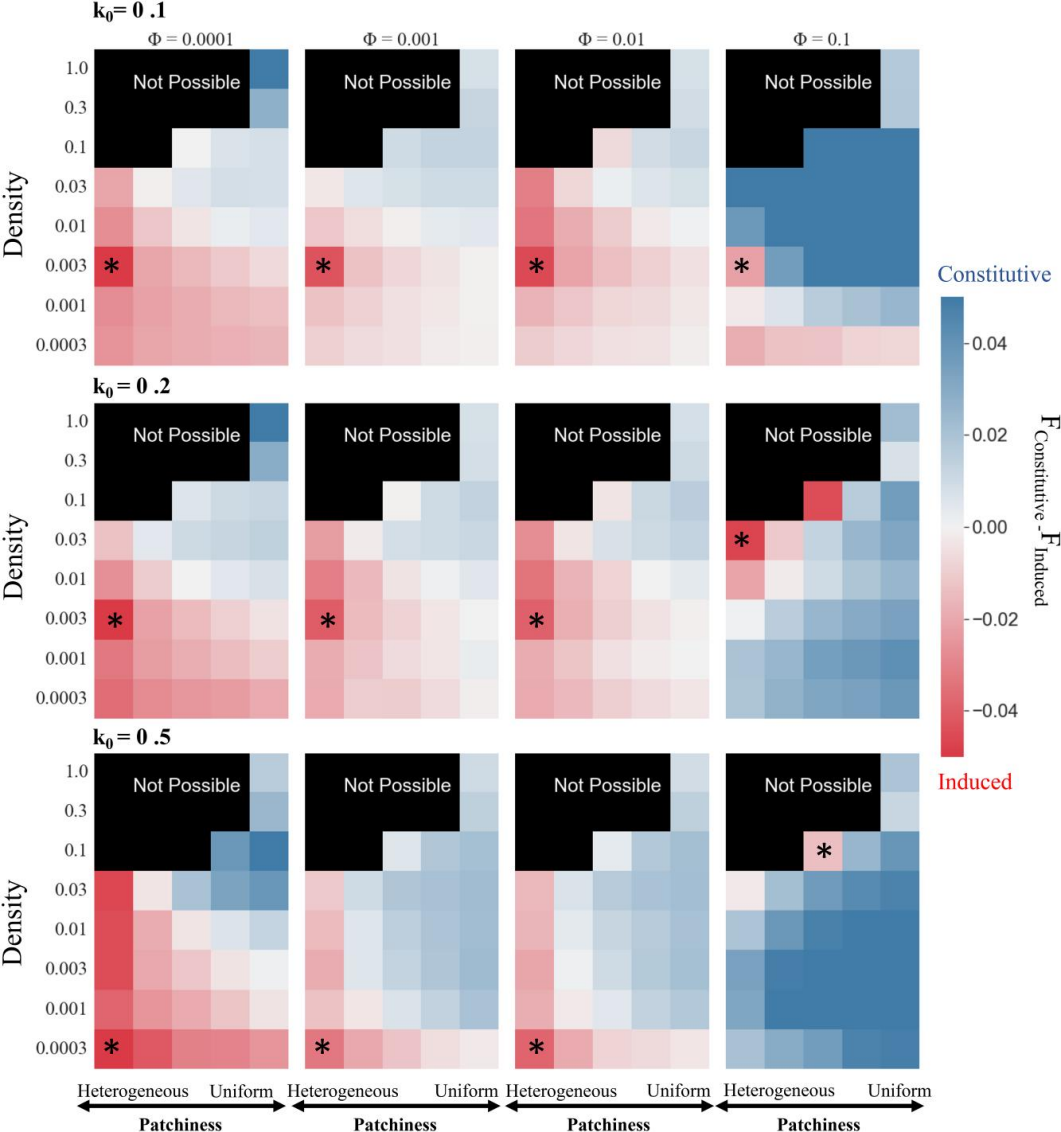
Induced responses tend to outperform constitutive defenses when flies inhabit environments with low densities and heterogeneous distributions of bacteria. The relative fitness of constitutive versus induced strategies is shown as heat maps for environments with different densities (*d*) and patchiness (*p*) of bacteria. Patchiness values (*p*) for each cell are reported in Supplementary Table 2. Red cells indicate that the fitness of the induced response (*F*_induced_) is higher than fitness of the constitutive defense (*F*_constitutive_), and blue cells indicate that *F*_constitutive_ > *F*_induced_. Heat maps in the same column show induced responses that were optimized with the same frequency of the sinusoidal input of bacteria (*Φ*), and heat maps in each row show the results for the same proliferation rate of bacteria ($k_{0}$). Asterisks show the environment in which the induced defense has the highest relative fitness.

To model unpredictable encounters, we compared the fitness of induced and constitutive defenses upon stochastic encounters with bacteria. To those ends, we simulated stochastic fly–bacteria interactions using random walks of a fly in a 100×100 grid with differing densities and patchiness of bacteria (Fig. 2). For most values of *Φ*, induced defenses had higher fitness than constitutive defenses in environments with a heterogeneous distribution and low-to-intermediate density of bacteria (Fig. 3). For the highest bacterial proliferation rate ($k_{0}$ = 0.5), induction had the highest relative fitness when bacterial density was lowest and distributed with maximal heterogeneity (Fig. 3). For other bacterial proliferation rates ($k_{0}$ = 0.1 and $k_{0}$ = 0.2), the relative fitness for induction was highest in environments with an intermediate density of bacteria and a heterogeneous distribution (Fig. 3). The only exception to these patterns was when the induced response was optimized with *Φ* = 0.1 and tested against bacteria with an intermediate ($k_{0}$ = 0.2) or a high ($k_{0}$ = 0.5) proliferation rate (Fig. 3). For these parameter combinations, induction performed best at higher bacterial densities. The poor performance of the induced defense optimized with *Φ* = 0.1 in the stochastic model is consistent with results from the sinusoidal model of fly–bacteria encounters (Supplementary Fig. 6 in File S1). We found that these results are robust to the choice of the fitness function and parameter values specifying protein degradation rates (refer to supplemental text and Supplementary Figs. 7–10 in File S1).

**Fig. 4.**
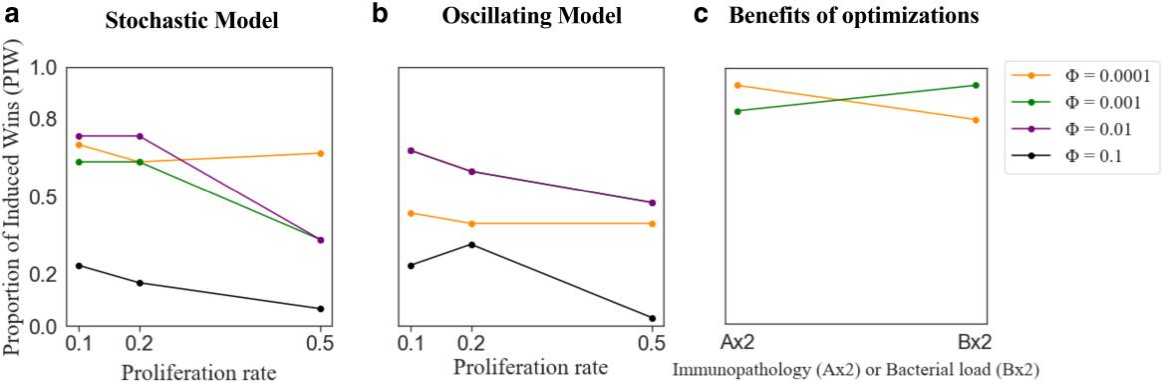
Proportion of induced wins (PIW) for different proliferation rates of bacteria and fitness functions. PIW (*y*-axis) was calculated using either the (A) stochastic or (B) sinusoidal oscillating model. a and b) Equation (10) was used to calculate fitness of the induced defense. c) PIW is plotted when either immunopathology or bacterial load has more impact on the host. A modified version of Equation (11) was used, where either A or B was multiplied by 2 (*x*-axis) and $k_{0}=0.1$. For results using the original Equation (11), refer to Supplementary Fig. 8 in File S1. a–c) PIW measures the proportion of environments in which an induced response outperforms constitutive defense. The results are shown for induced response optimized in environments with varying frequencies of encounters with bacteria (*Φ*). The green and purple lines (Φ=0.01 and Φ=0.001) on panel b overlap.

We further examined the effects of bacterial density, patchiness, and proliferation rate on the performance of the induced and constitutive defenses. To this end, we calculated the proportion of environments with different combinations of density and patchiness in which induction outperforms constitutive defense for a given bacterial proliferation rate and optimization of the induced response. This was done by calculating the fraction of red cells out of the total number of cells for each heat map in Fig. 3 for the stochastic model, and Supplementary Fig. 6 for the sinusoidal model, which we refer to as the “proportion of induced wins” (PIW). Under both models, induction tended to perform best (i.e. highest PIW) when bacterial proliferation rates were low or medium (Fig. 4a and b), and constitutive defense performed better (PIW decreased) as bacterial proliferation rates increased. This pattern is most pronounced for intermediate values of *Φ* (Φ=0.001 and Φ=0.01). The exception to this rule was when Φ=0.0001, in which case PIW remained fairly constant regardless of the bacterial proliferation rate. Induction performs worst (PIW is lowest) when optimized with Φ=0.1, especially when the bacterial proliferation rate is high.

Our analysis is unlikely to be biased by the parameters we selected in Figs. 3 and 4 because we compared the relative performance of induced and constitutive defenses across different values of *Φ* and $k_{0}$ using fixed values of density and patchiness. In other words, the relative performance (i.e. change in PIW) across proliferation rates (Fig. 4) should be robust to the parameters selected in the heat maps (Fig. 3). Because our results are consistent under both stochastic and sinusoidal models, we will proceed only with the stochastic model because it allows us to consider fitness differences between the induced and constitutive defenses when fly–bacteria encounters are unpredictable.

An optimized immune defense should ideally eliminate pathogens while minimizing immunopathology. To understand the benefits of different optimizations, we asked how different values of *Φ* effectively reduce the bacterial load and immunopathology (Fig. 4c). To this end, we used a fitness function that is dependent only on the amount of AMP and the bacterial load (Equation (11)). We optimized and compared induced and constitutive models when production of AMP has more impact on the host $\left(e^{-(2A\;+\;B)}\right)$ or when the bacterial load has more impact $\left(e^{-(A\;+\;2B)}\right)$. We did this for 2 values of *Φ* (0.0001 and 0.001). When induction was optimized with a low frequency of exposure to pathogens (Φ=0.0001), induction outperformed a constitutive defense in more environments (higher PIW) when the cost of immunopathology was greater than the cost of bacterial load. On the other hand, when optimized with a higher frequency of exposure (Φ=0.001), induction outperformed a constitutive defense in more environments if the cost of bacterial load was greater than immunopathology. Thus, different optimization approaches used here increase the effectiveness of induction by either reducing immunopathology or bacterial load.

### Inhabiting multiple types of environments favors induction

We next evaluated the performance of induced and constitutive defenses when a fly inhabits multiple environments. We were specifically interested in whether experiencing multiple types of environments (i.e. different bacterial densities and patchiness) favors induction, even when each individual environment favors constitutive defense. For that reason, we only sampled from environments in which constitutive defense outperforms induction (blue cells in Fig. 3), and then we measured the relative fitness of induced and constitutive defenses when flies inhabit multiple environments that were each sampled with equal probabilities. We calculated PIW when flies experience multiple bacterial environments for induced strategies that were optimized with different frequencies of bacterial input (*Φ*), across different numbers of possible environments, and at 3 different bacterial proliferation rates ($k_{0}=0.1,\;0.2,\;0.5$).

**Fig. 5.**
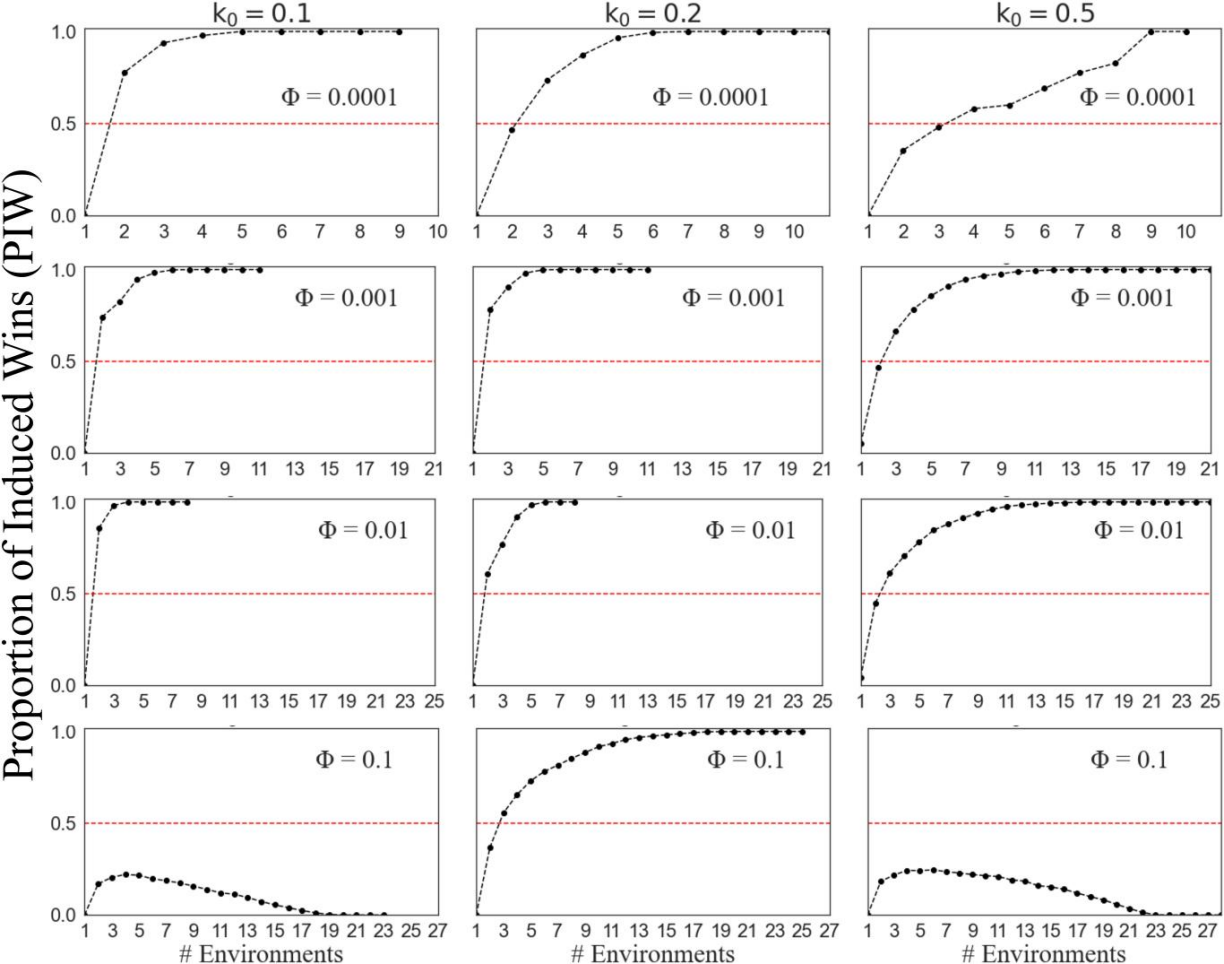
Induction outperforms constitutive defense as the number of different environments a fly inhabits increases. The proportion of times induction outperforms constitutive defense, PIW (*x*-axis), is plotted against the number of possible environments that are sampled (without replacement). Graphs in a column have the same bacterial proliferation rate ($k_{0}$), and graphs in a row have the same *Φ* value used to optimize the induced model. Induction outperforms constitutive defense above the dashed line (PIW > 0.5), and constitutive defense performs better below the dashed line (PIW < 0.5).

We found that inhabiting multiple environments favored induction over a constitutive defense. PIW generally increased as the number of environments increased, often reaching its maximum value of 1. Whether PIW reaches its maximum depends on the *Φ* used to optimize the induced defense. PIW reached the maximum for induced defenses optimized with lower frequencies of bacterial input (Φ=0.0001, *Φ* = 0.001, and *Φ* = 0.01), regardless of the bacterial proliferation rate (Fig. 5, first 3 rows). In addition, the rate of increase of PIW was greater for the lower proliferation rates ($k_{0}$ = 0.1 or 0.2) than the higher proliferation rate ($k_{0}$ = 0.5). PIW > 0.5 indicates that induction is favored because induced defenses win more than they lose. In general, PIW > 0.5 when the number of environments exceeded either 2 or 3, for most combinations of proliferation rates ($k_{0}$) and *Φ* values (Fig. 5). In contrast, when induction was optimized with a high frequency of bacterial input (Φ=0.1), PIW remained low for both low and high bacterial proliferation rates ($k_{0}$ = 0.1 or 0.5).

Next, we explored how the frequency of time the fly spends in 2 different environments affects the relative fitness of induced and constitutive defenses. As above, we calculated PIW when we sampled from individual environments where constitutive defense has a higher fitness than induction. We found that PIW was greatest when the 2 environments occur at equal frequencies (q=0.5), regardless of the frequency of the bacterial input used to optimize the induced response (Supplementary Fig. 11 in File S1). PIW decreased to 0 as the frequency of time spent in 1 environment increased.

### Negative regulators of Imd signaling affect the fitness of induction depending on the bacterial proliferation rate

To determine how negative regulators affect the fitness of induced immune responses, we compared the concentrations of 3 negative regulators of the Imd signaling pathway in response to bacteria with different proliferation rates ($k_{0}$) for induced defenses optimized with different frequency of exposure to bacteria (*Φ*). We focus on these parameters because the fitness of induced defenses depends on the bacterial proliferation rate and the value of Φ used to optimize the model. We selected induced defenses optimized with either Φ=0.0001 or Φ=0.01 because optimization with Φ=0.0001 offers the best defense against bacteria with a high proliferation rate (Fig. 4), and optimization with Φ=0.01 offers the best response against bacteria with a low proliferation rate (Fig. 4). We aimed to understand how the optimization of these different induced defenses shapes the production of negative regulators, thereby affecting their performance across bacterial environments.

**Fig. 6.**
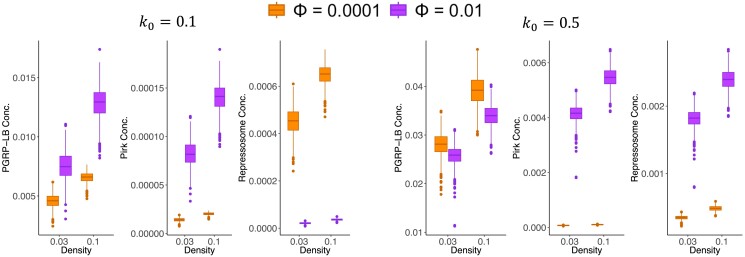
The concentration of proteins that act via negative feedback to modulate Imd signaling. The box plots show the concentration of proteins involved in negative regulation of the Imd signaling pathway (*y*-axis) in environments with 2 different densities (d) of bacteria (*x*-axis) that possess different proliferation rates ($k_{0}$) across 1,000 simulations. Induced defenses were optimized with different frequencies of bacterial encounters (Φ). The distribution of bacteria in all analyses is heterogeneous (p=1).

**Fig. 7.**
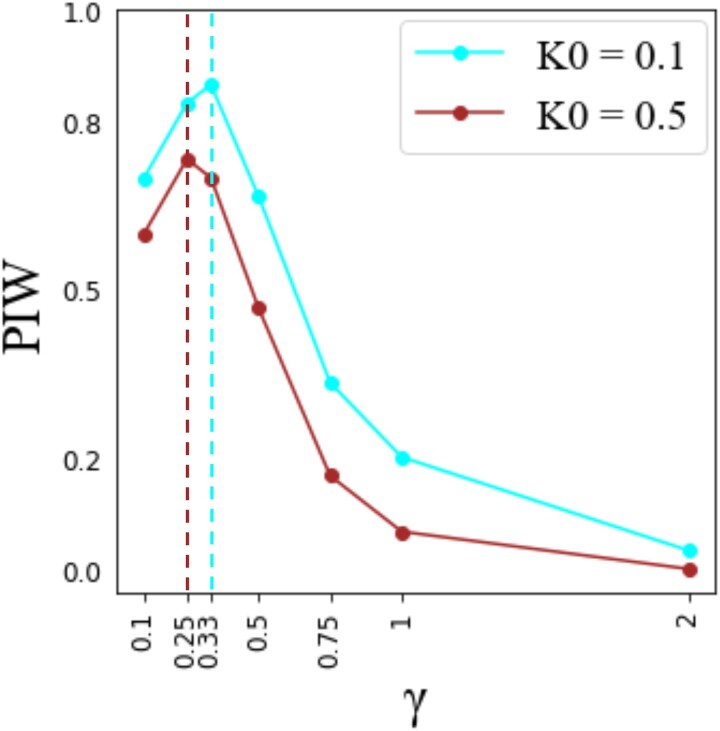
A higher rate of production of PGRP-LB and Pirk is beneficial against bacteria with a low proliferation rate. The proportion of induced wins (PIW) is shown (*y*-axis) for different parameter values that control the production rates of PGRP-LB and Pirk. The parameter values are changed by multiplying the optimized values for Φ=0.1 by a constant (γ) (*x*-axis). The analysis is done for low (cyan line) and high (brown line) bacterial proliferation rates (*k*_0_). The dashed lines delineate the *γ* value for which maximum PIW is achieved for low (cyan) and high (brown) bacterial proliferation rates.

We first explored the expression level of negative regulators when bacterial proliferation rates are low. When the bacterial proliferation is low ($k_{0}=0.1$), induction optimized with Φ=0.01 performs best (Fig. 4). This high fitness induced strategy produced more negative regulators that reduced input to the Imd pathway (PGRP-LB and Pirk) compared to an induced defense optimized with Φ=0.0001 (Fig. 6). Our results were consistent across different amounts of bacterial patchiness (Supplementary Fig. 12 in File S1). Therefore, we hypothesized that investing in negative regulators that reduce the input to the signaling pathway is beneficial against bacteria with low proliferation rates. We tested this hypothesis by measuring the fitness of induced defenses upon changing the parameter values that determine the production rates of PGRP-LB and Pirk. To this end, we first chose the induced defense with the worst performance, which is optimized by Φ=0.1 and $k_{0}$ = 0.5 (Figure 4). Next, we tried to improve the fitness of this induced defense by multiplying the 2 parameter values that determine the production rates of PGRP-LB and Pirk ($\beta_{3}$ and $\beta_{4}$ in Equations (6) and (7)) by a constant (γ). We measured improvement by calculating PIW upon changing the 2 parameter values (Fig. 7). We found that for a low bacterial proliferation rate, the maximum PIW is achieved for larger rates of production of PGRP-LB and Pirk (γ=0.33) compared to the high bacterial proliferation rate (γ=0.25 ) (Fig. 7). This is consistent with the hypothesis that the induced defense with a higher rate of production of PGRP-LB and Pirk is more successful against bacteria with a low proliferation rate.

**Fig. 8.**
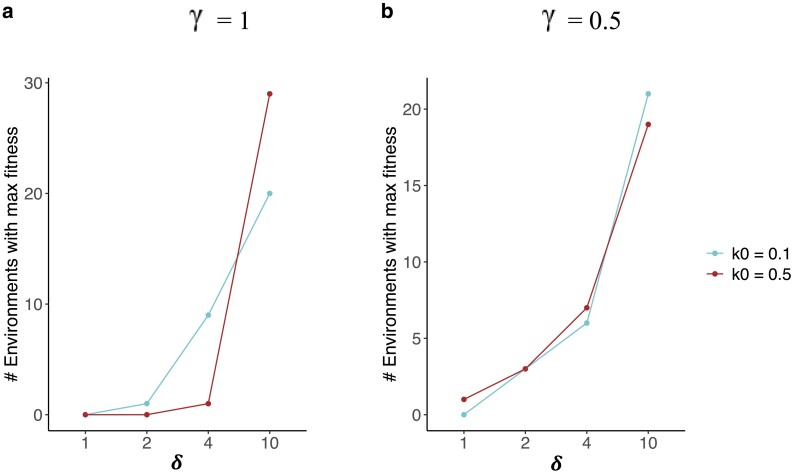
A stronger binding of the repressosome complex to the promoter is beneficial against bacteria with a high proliferation rate if PGRP-LB and Pirk have low expression. Number of environments (out of a total of 30) in which induced defense reaches its maximum fitness (*y*-axis) for 4 repressosome binding energy values (*x*-axis). The repressosome binding energy is manipulated by multiplying by a constant (δ) for an induced defense that is optimized with Φ=0.1 and $k_{0}$ = 0.5. The analysis is done for low (cyan line) and high (brown line) bacterial proliferation rates (*k*_0_). a) High production of PGRP-LB and Pirk (γ=1). b) Low production of PGRP-LB and Pirk (γ=0.5).

Next, we sought to understand what constitutes an effective immune defense against bacteria with a high proliferation rate. Because we found that higher rates of PGRP-LB and Pirk production were detrimental against bacteria with a high proliferation rate (Fig. 7), we focused our attention on the third negative regulator, namely, the repressosome complex. For the high bacterial proliferation rate $\left(k_{0}=0.5\right)$, the induced defense optimized with Φ=0.0001 performed best in the stochastic model and well in the oscillating model of bacterial exposure (Fig. 4). This optimization produced less repressosome than induction optimized with Φ=0.01 (Fig. 6). However, in our model, the fitness of an induced defense is influenced by both the production rate of the repressosome and its binding energy. Therefore, we needed to look beyond the concentration of repressosome to measure its effect on fitness. Indeed, the binding energy for repressosome was lower (a more effective binding) for induction optimized with Φ=0.0001 ($Z_{s}$ = 5 as opposed to $Z_{s}$ = 5.1 for Φ=0.01). We then tried to improve the immune response optimized with Φ=0.1 and $k_{0}\;=0.5$ by multiplying the binding energy of the repressosome complex ($Z_{s}$ in Equation (9)) by a constant (*δ*). We did not change the rate of repressosome production because by manipulating the binding energy of the repressosome, we could measure the effect of repressosome on fitness without the confounding effect of the cost of the production of the repressosome itself. We found that the PIW was consistently reduced for larger binding energy values (poor binding) regardless of the bacteria proliferation rate (Supplementary Fig. 13 in File S1). Hence, assessing PIW does not provide insight into the potential benefits of repressosome upon induction. We next measured the fitness of induced defenses in individual environments with a unique combination of bacterial density and patchiness upon changing the binding energy of the repressosome complex. We then identified the binding energy value that maximized fitness across 4 distinct binding energy values within each environment (δ=1,2,4,10). Contrary to our expectation, we found lower repressosome binding energy to be beneficial against bacteria with a low proliferation rate $\;(k_{0}=0.1)$ rather than a high proliferation rate $\left(k_{0}=0.5\right)$. Specifically, we found that the induced defense reached its maximum fitness across more environments for lower binding energy values (e.g. δ= 2 or 4) when the bacterial proliferation rate was low $\left(k_{0}=0.1\right)$ than when it was high $\;(k_{0}=0.5)$ (Fig. 8a). However, when we reduced the rate of PGRP-LB and Pirk production (γ=0.5), we observed the opposite pattern (Fig. 8b). For lower production rates of PGRP-LB and Pirk, the induced defense reached its maximum fitness across more environments for lower binding energy values (e.g. δ=1) when the bacterial proliferation rate was high $\left(k_{0}=0.5\right)$ than when it was low $\left(k_{0}=0.1\right)$ (Fig. 8b). Thus, our results suggest that damping the immune response with decreasing repressosome binding energy is effective against bacteria with a high proliferation rate if PGRP-LB and Pirk are produced at low levels (Fig. 8b).

## Discussion

We modeled constitutive and induced immune defenses of *D. melanogaster* (Fig. 1) to determine the conditions in which each was favored. To those ends, we compared the relative fitness of induced and constitutive defenses in environments with different densities and patchiness of bacteria (Fig. 2). Our model predicts that induction is favored in environments where bacterial exposure is uncertain, which can be caused by heterogeneous distributions of bacteria (Fig. 3) or fluctuations in bacterial density and patchiness (Fig. 5). We also found that induction is favored when flies experience multiple bacterial environments, even when those individual environments favor a constitutive defense in isolation (Fig. 5, Supplementary Fig. 11 in File S1). Our model did not explore the evolution of the immune network across generations using, for example, a population genetics approach because it was not tractable to do given the realistic mechanistic details we included. By including multiple levels of negative regulation in our model, we found that investing in negative regulators that reduce the input to the system results in an effective defense against bacteria with low proliferation rates (Fig. 7). On the other hand, our model predicts that investing in negative regulators that reduce the output (AMP) produces effective defense against bacteria with a high proliferation rate (Fig. 8). Thus, our results provide unique insights into the evolution of negative feedback mechanisms that control immune responses and other induced defenses across animals and plants (Mukherjee *et al*. 2008; Arimoto *et al*. 2018; Rothschild *et al*. 2018; Li *et al*. 2022).

### Uncertainty favors an induced immune response

**Fig. 9.**
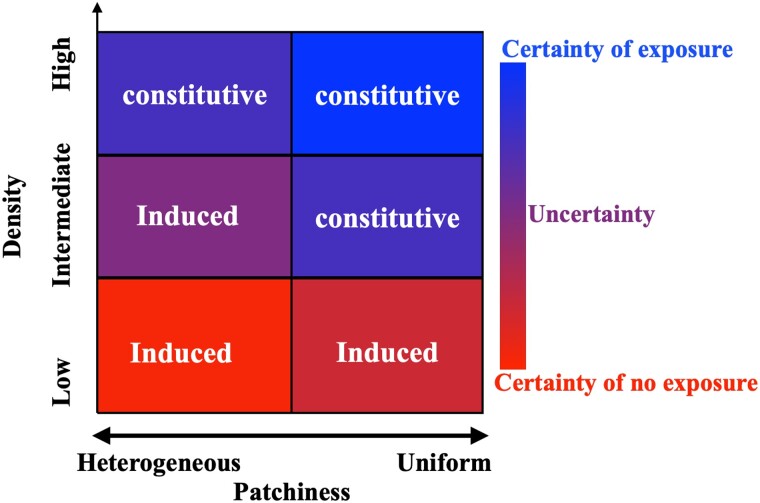
Certainty and uncertainty of encountering bacteria in environments with different density and patchiness of bacterial populations. Certainty of exposure is shown with shades of blue, and certainty of no exposure is shown with shades of red. Environments that favor constitutive defenses are specified by “constitutive”, and environments that favor induced defenses by “induced”.

Although uncertainty of infection is inherent to every host–pathogen system, the interactions between different sources of uncertainty have not been explicitly studied. In our model, uncertainty of bacterial exposure can come from multiple sources, and we find that the interactions of these different sources can affect the extent to which uncertainty favors induction. For example, the distribution of bacteria in the environment can create uncertainty, which favors induction in specific combinations of bacterial densities and patchiness. The uncertainty of exposure is maximal when bacteria are at intermediate densities with heterogeneous distributions (Fig. 9). In these environments, there are likely to be long periods in which bacteria are not encountered (because of their heterogeneous distribution), combined with bursts of high exposure (because they are intermediate density). This combination increases the relative fitness of induction compared to a constitutive defense (Fig. 5) because an induced defense only produces AMPs during the bursts of high exposure, as opposed to a constitutive defense that produces unnecessary AMPs when there are no encounters with pathogens. In addition, while a constitutive strategy that produces a low level of AMP may reduce costs, it would be insufficient to eliminate pathogens when a host enters a patch of bacteria (relative to the induced strategy).

Uncertainty in our model can also come from inhabiting multiple environments with different combinations of bacterial densities and patchiness. A key finding of our model is that induction is favored when flies experience multiple environments, even when a constitutive defense is favored in each individual environment (Fig. 5 and Supplementary Fig. 11 in File S1). Even though it is well established that uncertainty favors induction, our model is unique in predicting that induction can be favored upon environmental fluctuations even if constitutive defense outperforms induced defense in each individual environment.

There are important parallels between the uncertainty that arises from multiple possible environments in our model and selection pressures that act on AMP protein sequences. Balancing selection can maintain sequence variation in *Drosophila* AMP genes (Unckless and Lazzaro 2016). Balancing selection on AMPs likely occurs as a result of fluctuations of bacterial communities in environments inhabited by flies (Abdul-Rahman *et al*. 2021). Such fluctuating environments are similar to those that favor induced defenses in our model. Therefore, uncertainty might have been responsible for both the prevalence of induced expression of AMPs and AMP sequence variation in *Drosophila*.

### Bacterial proliferation rate affects the benefits of induction and negative regulators

We found that the effect of bacterial proliferation rates on host fitness varied depending on bacterial density and distribution. While a previous model addressed the uncertainty surrounding the proliferation rate of bacteria encountered by the host (Hamilton *et al*. 2008), our model uniquely enabled both individual and combined investigations into bacterial proliferation rate and uncertainty. We found that when bacterial density was intermediate and the distribution was heterogeneous, induced defense had higher fitness against bacteria with low proliferation rates (Fig. 3). Conversely, in environments with low bacterial densities and heterogeneous distributions, higher bacterial proliferation strongly favored induced defense (Fig. 3). These results emerge because low-density environments rarely result in fly–bacteria interactions, necessitating a strong immune response only when proliferation rate is high. Constitutive AMP expression is not favored in these low-density environments because the cost outweighs the benefits due to the low frequency of encounters with pathogens.

Our model also predicts that bacterial proliferation rate affects the benefits of producing different negative regulators of the Imd pathway. This is a significant step in understanding the evolution of immune response because it links 2 pervasive phenomena in immunology: the existence of diverse negative feedback mechanisms operating at various stages of signaling, and the diversity of bacterial species encountered by the host within its environment. Specifically, we found that production of negative regulators that reduce the input into the immune signaling pathways, such as Pirk and PGRP-LB in the Imd signaling pathway, increases the relative performance of an induced response against bacteria with low proliferation rates (Fig. 7). We hypothesize that investing in negative regulators that reduce the input into the system can rapidly shut down the pathway and prevent wasteful investment in immunity. This is an effective strategy against bacteria with low proliferation rates because there is not an urgent need to produce a rapid response to a slowly replicating pathogen. In contrast, production of negative regulators that function downstream of signaling to reduce the output—such as the repressosome complexes, which compete with Relish (the transcription factor that induces AMP expression in the Imd pathway) for binding to the promoter of AMP genes—results in an effective immune response to bacteria with a high proliferation rate (Fig. 8). When defending against bacteria with a high proliferation rate, investing in negative regulators that reduce the output (AMPs) is more effective because it lowers the cost of AMP production without entirely shutting down the pathway. Unlike PGRP-LB and Pirk, the repressosome does not reduce the concentration of active Relish. In this way, active Relish is available for a timely response upon subsequent encounters with rapidly proliferating pathogens. When bacterial proliferation is high, stopping the exponential growth of bacteria while minimizing the investment in immunity significantly impacts host fitness. Previous theoretical work that did not simultaneously model the multiple negative feedback loops and bacterial proliferation rates could not have predicted these results. For example, Ellner *et al*. (2021), who provided a detailed model of the Imd pathway, did not explicitly model the action of the 3 negative feedback loops (PGRP-LB, Pirk, and the repressosome complex) and therefore could not have observed their different effects. Future empirical studies could test how negative regulators that function at different steps of signaling affect the survival of hosts when they encounter bacteria with different proliferation rates.

The finding that negative feedback mechanisms operating at different steps of the signaling pathway are beneficial against bacteria with different proliferation rates suggests that uncertainty not only favors induction but also shapes the mechanisms that regulate induction. In the natural environment, animals and plants encounter an array of bacteria that multiply at varying rates (Duneau *et al*. 2017). Thus, the evolution of immune responses is not only influenced by the unpredictability of bacterial encounters but also by uncertainty with regard to rates of bacterial proliferation. This might explain the fact that immune responses are often regulated at different steps in the signaling pathway. For example, interferon signaling includes downregulation of cell surface receptors and inhibition of downstream transcription factors (Porritt and Hertzog 2015). Our study contributes to understanding the prevalence of negative regulators with distinct functions in immune signaling pathways. In addition, given the widespread prevalence of negative feedback loops beyond immune signaling pathways, the insights derived from this model could potentially extend to other signaling pathways as well (Perrimon and McMahon 1999; Yu *et al*. 2008; Shilo 2014; El-Samad 2021). Future work should further evaluate how different types of negative feedback are favored in relation to the uncertainty of threats to a host.

## Supplementary Material

jkae182_Supplementary_Data

## Data Availability

All code is available from https://github.com/danialasgari/Uncertainty-favors-an-induced-immune-response-to-infection-. Supplemental material available at G3 online.
