# Supplementary material for: The role of uncertainty and negative feedback loops in the evolution of induced immune defenses
Source: G3 (Bethesda). 2024 Aug 6;14(10):jkae182. doi: 10.1093/g3journal/jkae182 (PMC11457078; doi:10.1093/g3journal/jkae182)
Supplement: jkae182_Supplementary_Data [file jkae182_supplementary_data.pdf]

Simulation of the bacterial environment

We simulated bacterial populations on a 2-dimensional lattice (100×100) using a simple random walk algorithm with periodic boundary conditions (Figure 2). We placed bacterial colonies at positions on the lattice which were determined according to a random walk. We initialized the walk at a random starting coordinate [i_0, j_0], and the subsequent direction of movement was specified to be left [$i_0 - p, j_0$], right [$i_0 + p,\ j_0$], up [$i_0, j_0 + p$], or down [$i_0, j_0 - p$], with equal probabilities ($\frac{1}{4}$), where p describes the (constant) step size. A bacterial colony was then placed on the lattice at the updated coordinate [i, j] . If a lattice point was already occupied by a bacterial colony, the lattice was not updated, the colony count remained unchanged, and another step was taken. The process was repeated until a predetermined number of colonies had been added to the lattice. The number of colonies added to the lattice determined the density of bacteria ($d\ =\ \frac{\#colonies}{100\times100}$) in the environment. The value of the random walk step size, p, determines the patchiness of the bacterial distribution; low values of p create a more heterogeneous distribution of bacteria, while high values create uniform distributions (Figure 2).

Movement of the fly through the environment

The random walks were performed as described above for seeding the lattice, but all fly walks used step size p =1. When a fly landed at a given coordinate [i,j], the immune response was determined by the input function $f(t)$ (Eq. 1), such that, if a bacterial colony was present $f(t)$=1, and $f(t)$=0 otherwise. Thus as the fly moves through the environment $f(t)$ takes values 1 or 0 depending on the presence or absence of bacteria at the current lattice position. This process simulates stochastic fly-bacteria interactions. We solved the system of ODEs using the Runge-Kutta Method (RK4) algorithm for the random walk model.

Optimization of the induced model

We used the Muller (1959) method to generate random points in an 11-dimensional space, where step size = 0.01. First, we found the position vector of a point (u) in an 11-dimensional space by generating 11 independent normal deviates (Eq. ES1).

$$u_i\ =\ N(0, 0.01);\ i\ =\ 1,\ldots, 11 \qquad \text{(ES1)}$$

The length of the vector is calculated using Equation (ES2).

$$w = \sqrt{\sum_{i=1}^{11} u_i^2} \quad \text{(ES2)}$$

The updated value for each variable (X') is found by summing the current value (X) with the direction cosines ($\frac{u_i}{w}$) multiplied by the step size (0.01), using Equation (SE15).

$$X_i^{'} = \left| 0.01\, (\frac{u_i}{w}) + X_i \right| \quad \text{(SE3)}$$

We used Equation (10) to calculate the fitness of induced defense ($F_{Induced}$) using the 11 parameters as input. At each step in the optimization walk, the 11 parameters are simultaneously updated. If $F_{\lambda'_2,\lambda'_3,..,Z'_s} > F_{\lambda_2,\lambda_3,..,Z_s}$, the new set of parameter values is accepted; otherwise, the set is rejected. We used 10,000 steps for the lower bacterial proliferation rates ($k_0 = 0.1$ and $k_0 = 0.2$), and 20,000 steps for the higher proliferation rate ($k_0 = 0.5$) because it took longer for fitness to plateau for the higher proliferation rate.

Random walks within an 11-dimensional landscape are computationally intensive if $f(t)$ is stochastic. To perform a more efficient search, we calculated fitness at each step of our random walk using a deterministic oscillating input with different frequencies to the system of ODEs instead of a stochastic one ($f(t) = sin(t\ \Phi)^2$). We optimized the induced model using different values of Φ, i.e., the frequency of the sinusoidal input. Higher Φ describes a higher density of bacteria. Using a sinusoidal input is more efficient than stochastic simulations of bacterial distributions at each step of the walk through parameter space, and it also dispenses with the need for simulating random walks of flies among bacteria at each step. Different local optima might be reached when starting from different points in the landscape. Therefore, we considered a large and a small starting value for each parameter and ran a random walk for every combination of large and small parameter values. This makes a total of 2,048 walks for the purpose of optimizing the 11-parameter model ($2^{11} = 2048$).

To test for the robustness of the assumption of identical degradation rates (λ_2) in the 11-parameter model, we also considered a model with different degradation rate parameters for R, N, L, P, S, and A. This created a 16-parameter model. Optimizing this model requires a total of 65536 (2^{16}) random walks, which is 5 orders of magnitude more computationally intensive than the 11-parameter model. To overcome this problem, we optimized the 16-parameter model using two approaches. In the first approach, we considered a large and a small starting value for the six degradation rates and started the other 10 parameters at their optimum values in the 11-parameter model. In the second approach, we also considered a large and a small starting

value for the six degradation rates but fixed the other 10 parameters at their optimum values in the 11-parameter model. Both approaches entail a total of 64 (2^6) walks for the purpose of optimizing the 16-parameter model, following optimization of the 11-parameter model.

Optimization of PG production and degradation rate

We evaluated how the rate of bacterial PG production affects the performance of an induced immune response across different frequencies of bacterial exposure. We optimized induced defenses for four rates of bacterial PG production (α = 0.2, 1, 2, and 4; Figure S3A). Our results were qualitatively similar across different α and Φ values (Figure S3B). Induction performs best when bacterial density is intermediate and the distribution is heterogeneous, regardless of the α and Φ values (Figure S3). We only focused on results for $\alpha = 2$ because it maximizes the performance of induced defenses when flies encounter two different bacterial distributions (Figure S3C).

We chose a small value for the natural degradation rate of PG ($\lambda_1 = 0.01$) because the natural degradation rate is much slower compared to degradation by PGRPs (Filipe, Tomasz, and Ligoxygakis 2005). We found that, when $\lambda_1 = 0.01$, the parameter controlling production of PGRP-LB (β_3) is larger than λ_1 (Figure S4A), consistent with more degradation by PGRP-LB than the natural degradation rate. However, when $\lambda_1 = 0.05$, β_3 is smaller (Figure S4B). Therefore, setting λ_1 to 0.01 is biologically realistic because it captures the important role that PGRP-LB plays in degradation of PG. By setting $\alpha = 2$ and $\lambda_1 = 0.01$, we found that increasing the frequency of fly-bacteria encounters (Φ) reduced the optimal fitness of the induced response, regardless of the bacterial proliferation rate (k_0) inside the fly (Figure S5). Our results therefore suggest that induced responses are less fit in environments with a higher frequency of fly-bacteria interactions.

Intermediate bacterial density and heterogeneous distribution favor an induced defense regardless of the choice of the fitness function and protein degradation rates.

Next, we evaluated how the fitness function affects the relative performance of the induced and constitutive strategies. To this end, we optimized the induced defense by assuming that the production of proteins involved in Imd signaling is not costly, and the only cost comes from bacterial proliferation and production of AMPs, perhaps due to the immunopathologic effect of AMP expression (Eq. 11; Figure S7). This assumption increases the relative fitness of

the induced defense, such that induction generally outperforms constitutive defense regardless of the bacterial environment (Figure S8A). Nonetheelss, consistent with our results using a more costly fitness function (Figure 3), induced defense has the highest relative fitness in environments with intermediate densities and heterogeneous distributions of bacteria (Figure S8A). Also, the relative fitness of induction is higher in heterogeneous and low density environments, and induction performs worst in high density and uniform environments, regardless of the fitness function (Figure 3; Figure S8A). As before, induction performs worst when optimized with $\Phi = 0.1$ (Figure S8B). Because we observe the same overall pattern of relative fitness of induced and constitutive strategies using both fitness functions, we conclude that our results are robust to the choice of the fitness function. For the remainder of the manuscript, we use the fitness function in which Imd signaling is costly (Eq. 10).

When calculating the fitness of an induced defense we assume identical degradation rates (λ_2) for Imd signaling pathway proteins (it is an 11-parameter model), and we tested the effects of this assumption. To that end, we optimized a model of induction with different parameters for degradation rates (16-parameter model). We used two approaches to optimize the 16-parameter model, both of which start from the parameter values optimized in the 11-parameter model. In the first approach, we search for the optimum fitness by allowing small changes for all 16 parameters. Using this approach we found that the six degradation rate parameters converge to the single degradation parameter in the 11-parameter model (λ_2) (Figure S9). This suggests that there is no benefit to separately optimizing each degradation rate. In the second approach, we found the optimum induced defense by varying the six degradation parameters, while fixing the other 10 parameters. Using this approach we found optimized induced defenses with different degradation rates for proteins involved in Imd signaling (Figure S9). We found that the induced response has the highest performance when compared to constitutive defense under the same conditions for both the 11-parameter and 16-parameter models (Figure S10). We therefore conclude that the 11-parameter model is sufficiently complex to capture the costs and benefits of an induced immune response.

References:

Filipe, Sergio R., Alexander Tomasz, and Petros Ligoxygakis. 2005. “Requirements of Peptidoglycan Structure That Allow Detection by the Drosophila Toll Pathway.” *EMBO Reports* 6 (4): 327–33.

Muller, Mervin E. 1959. “A Note on a Method for Generating Points Uniformly on N-Dimensional Spheres.” *Communications of the ACM* 2 (4): 19–20.

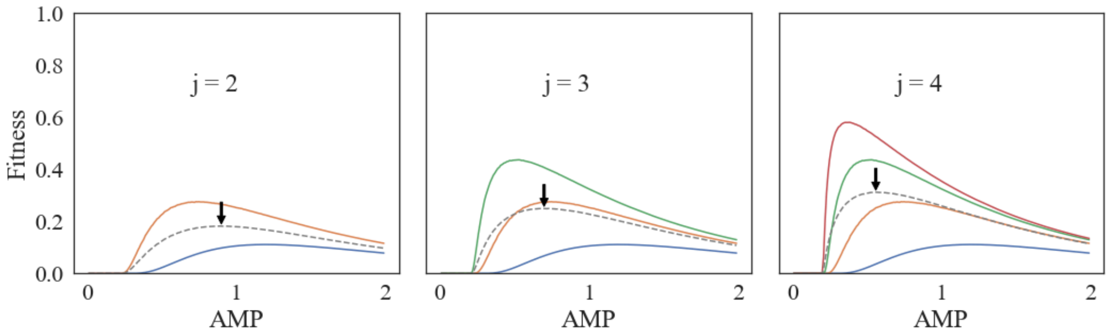

Figure S1. Finding the best constitutive defense when a fly inhabits j environments, with different density and patchiness of bacteria, with equal probability. Fitness is shown on the Y-axis in environments with different density and/or patchiness of bacteria (different colors) for varying levels of constitutive AMP expression (X-axis). The dashed line represents the fitness if the fly randomly encounters j environments each with a probability $\frac{1}{j}$. The arrow shows the optimum fitness upon random encounter with j environments.

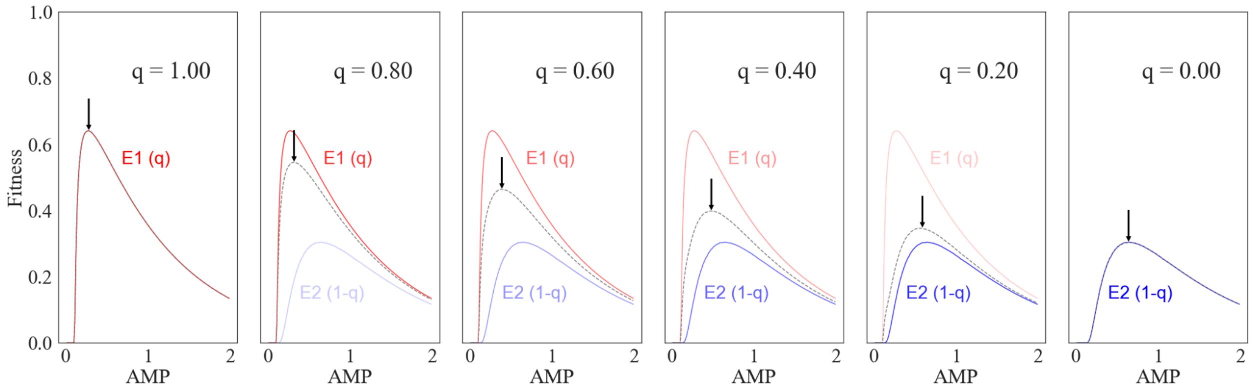

Figure S2. Optimum fitness of constitutive defense (shown with an arrow) when flies inhabit two environments, E1 and E2, with probability q or $1 - q$, respectively. Red lines represent the fitness in E1 and blue lines represent the fitness in E2. The dashed line is the fitness when the fly inhabits both environments with different probabilities. The optimum fitness is shown with an arrow.

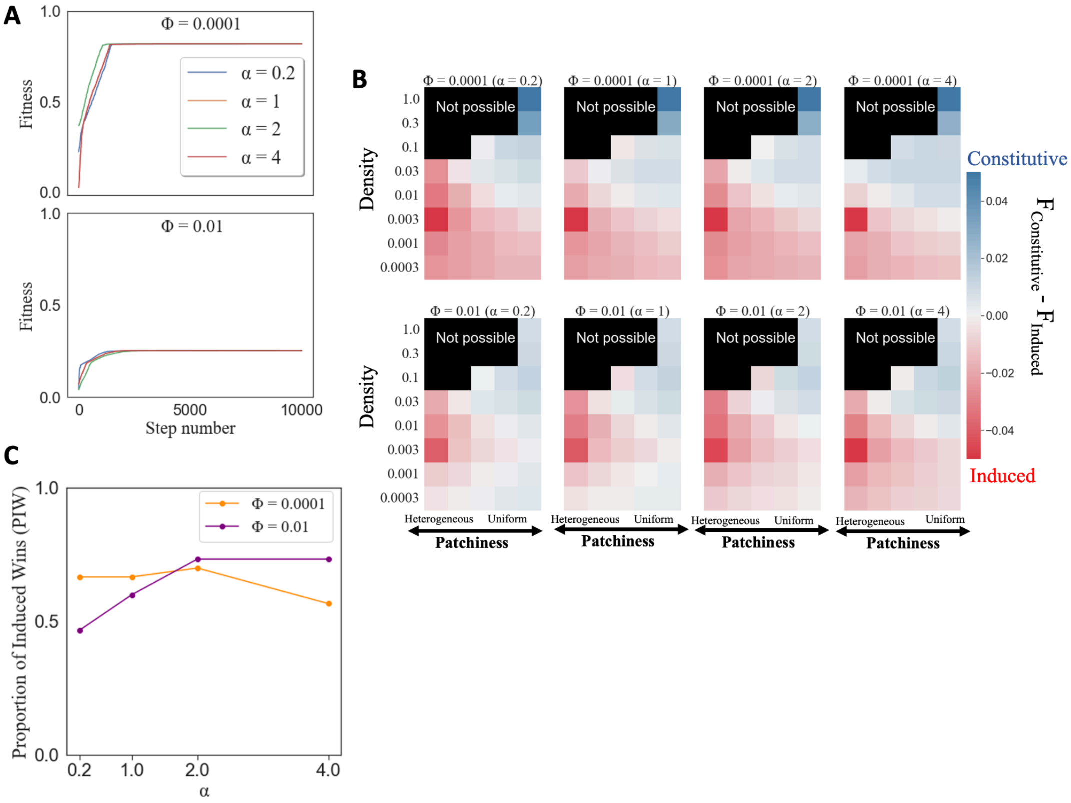

Figure S3. Results for optimization of induced defense for four values of α (0.2, 1, 2, and 4). **A:** The fitness value (Y-axis) at every step of optimization (X-axis) for optimization that resulted in the highest fitness amongst 2048 simulations. Optimizing induced defense for different values of α does not change the optimum fitness value for two frequencies of input to the system ($\Phi = 0.0001$ and $\Phi = 0.01$). **B:** The relative fitness of induction vs constitutive defense (color bar) in environments with different patchiness (X-axis) and bacterial density (Y-axis). Induction has a higher fitness in red-colored cells than the best constitutive defense. The highest relative fitness is shown with an asterisk. Induction is preferred in heterogeneous environments with low bacterial density regardless of the value of α. **C :** Comparing the proportion of induced wins (Y-axis) across different values of α (X-axis) for two values of Φ. The proportion of induced wins is highest for $\alpha = 2$ regardless of the value of Φ.

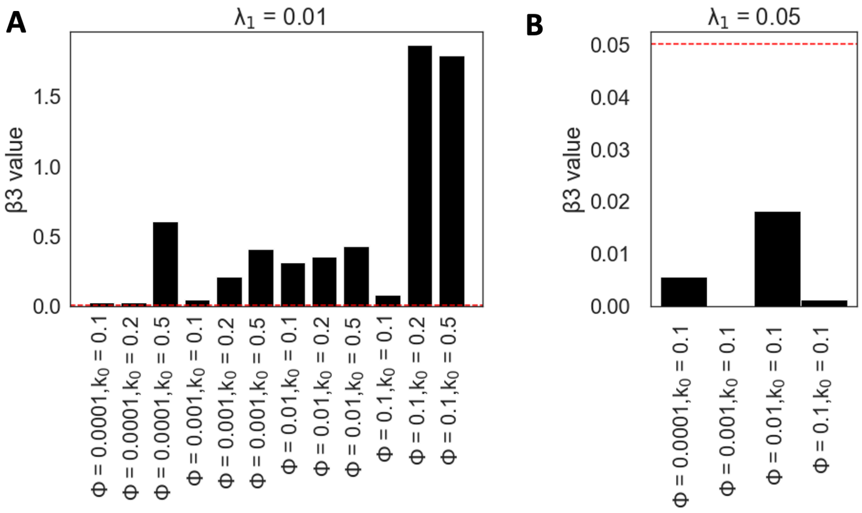

Figure S4. Optimization of the induced defense with two values of λ_1. The X-axis are the parameters affecting the production of PGRP-LB (β_3), and the Y-axis is the value of β_3 in the optimized model. **A:** Optimization of the induced defense with $\lambda_1 = 0.01$ using different values of Φ and k_0. The red dashed line is the value of λ_1 (0.01). **B:** Optimization of the induced defense with $\lambda_1 = 0.05$ using different values of Φ. The red dashed line is the value of λ_1 (0.05).

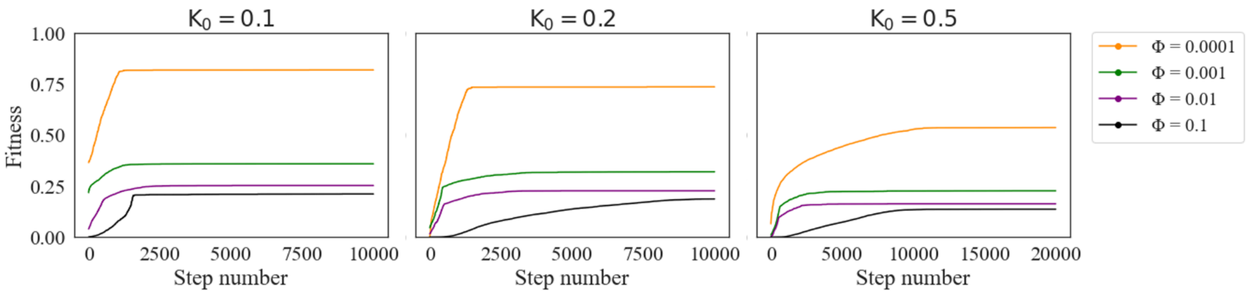

Figure S5. Optimization of the induced response using an oscillating deterministic input (sinusoidal) with different frequencies (Φ) for three different proliferation rates of bacteria inside the fly (k_0) and $\alpha = 2$. Optimization is performed by taking 10,000 steps in the fitness landscape for low proliferation rates ($k_0 = 0.1$ and 0.2) and 20,000 for the high proliferation rate ($k_0 = 0.5$). The X axis is the number of steps taken in the fitness landscape.

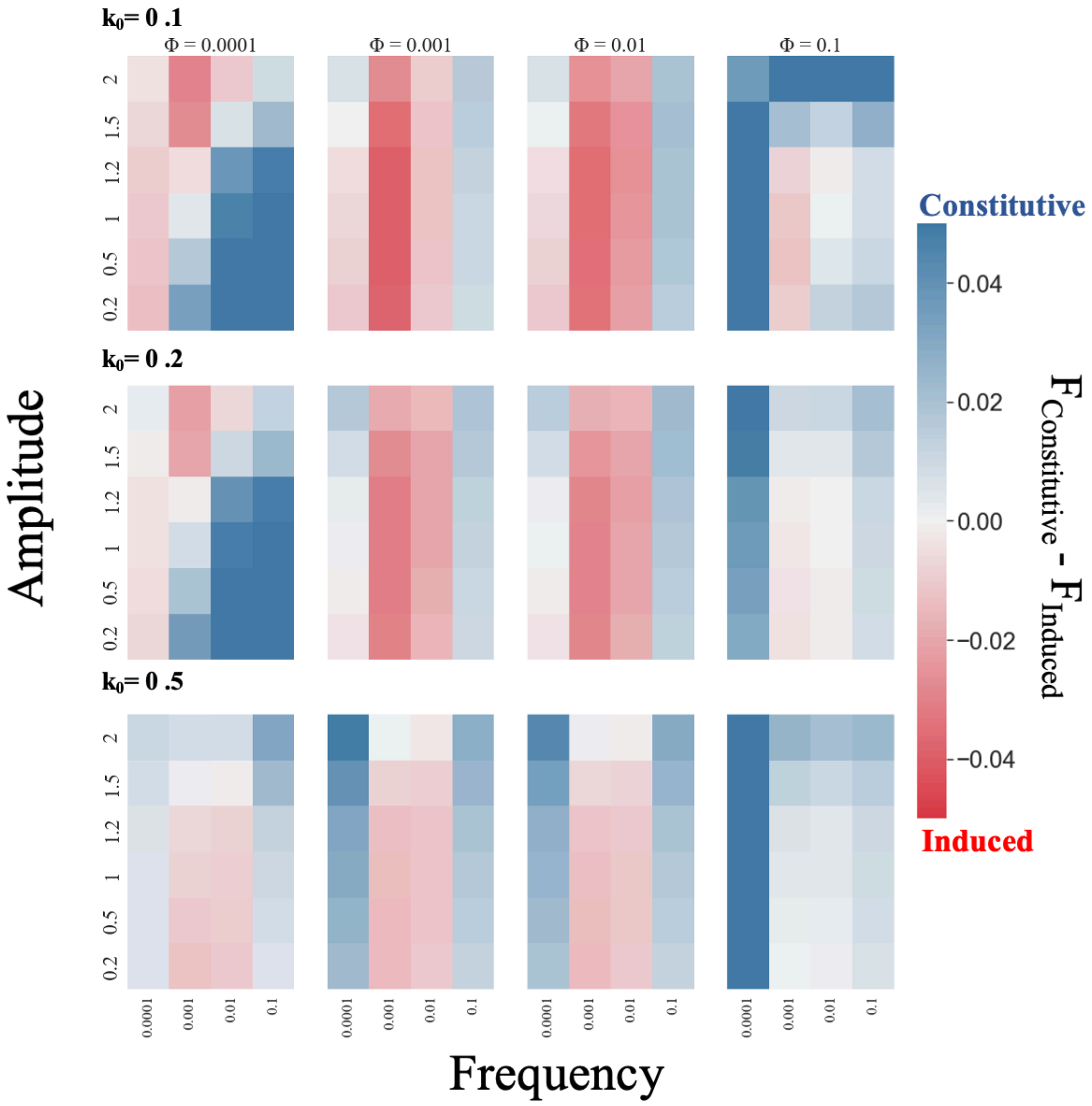

Figure S6. Induced responses tend to outperform constitutive defenses when flies encounter bacteria with intermediate frequencies. The relative fitness of constitutive versus induced strategies is shown as heatmaps for environments with different amplitudes and frequencies of sinusoidally oscillating encounters with bacteria. Red cells indicate that the fitness of the induced response ($F_{Induced}$) is higher than the fitness of the constitutive response ($F_{Constitutive}$), and blue cells indicate that $F_{Constitutive} > F_{Induced}$. Heatmaps in the same column show induced responses that were optimized with the same frequency of the sinusoidal input of bacteria (Φ), and heatmaps in each row show the results for the same proliferation rate of bacteria (k_0).

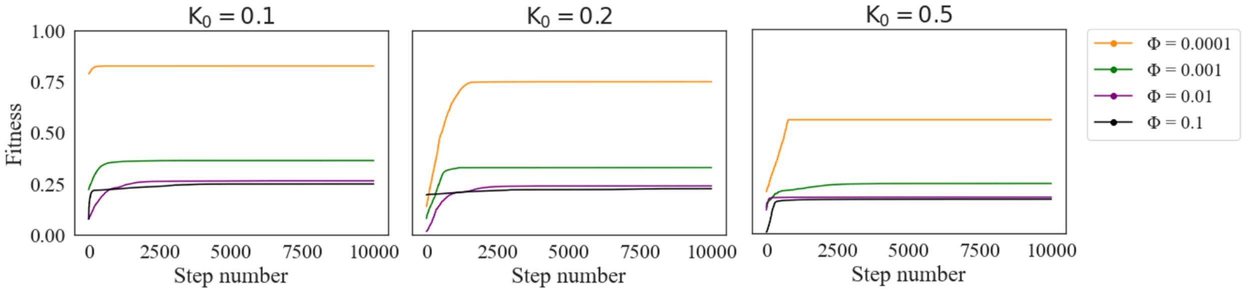

Figure S7. Optimization of the induced response under the assumption that production of signaling proteins does not affect the fitness. Induced defense is optimized using an oscillating deterministic input (sinusoidal) with different frequencies (Φ) for three different proliferation rates of bacteria inside the fly (k_0) and $\alpha = 2$. Optimization is performed by taking 10,000 steps in the fitness landscape. The X axis is the number of steps taken in the fitness landscape.

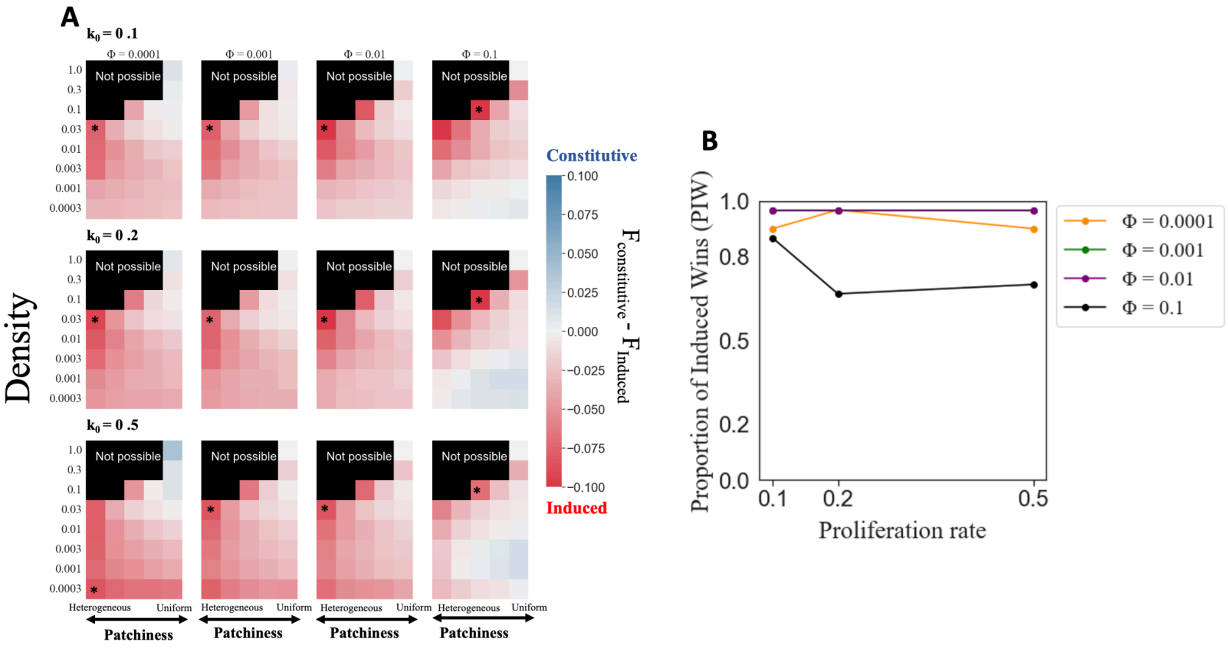

Figure S8. Results for comparison of constitutive and induced defenses, assuming that production of signaling proteins does not affect fitness. **A.** The relative fitness of constitutive vs induced defense (color bar) in environments with different patchiness (X-axis) and bacterial density (Y-axis). Induction has a higher fitness in red-colored cells than the best constitutive defense. Induction is preferred in heterogeneous environments with low bacterial density regardless. The highest relative fitness is shown with an asterisk. Heatmaps in the same column show induced responses that were optimized with the same frequency of the sinusoidal input of bacteria (Φ), and heatmaps in each row show the results for the same proliferation rate of bacteria (k_0). **B.** The proportion of induced wins (number of red cells divided by the total number of cells) is graphed for different optimizations of the induced defense.

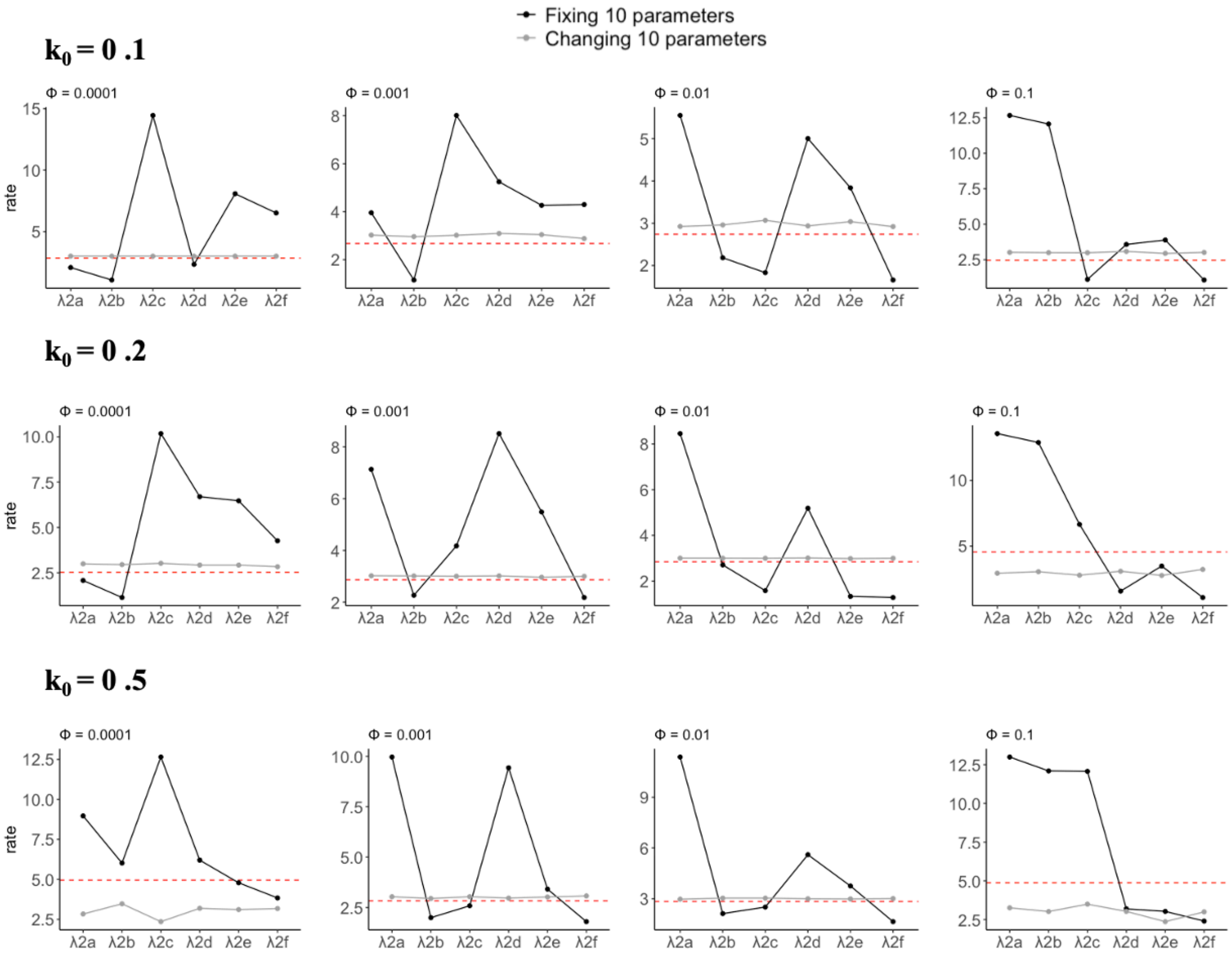

Figure S9. Degradation rate (Y axis) for proteins involved in Imd signaling: R ($\lambda2_a$), N ($\lambda2_b$), L ($\lambda2_c$), P ($\lambda2_d$), S ($\lambda2_e$), and A ($\lambda2_f$) (X-axis) are shown for two methods of optimization of the induced defense (columns) against different bacterial proliferation rates (rows). The black line shows the optimized degradation rates when the other 10 parameters are fixed during optimization. The gray line shows optimized degradation rates when all 16 parameters are allowed to fluctuate during optimization. The red dashed line is the optimized degradation rate under the assumption of identical degradation rate (λ_2) for all proteins.

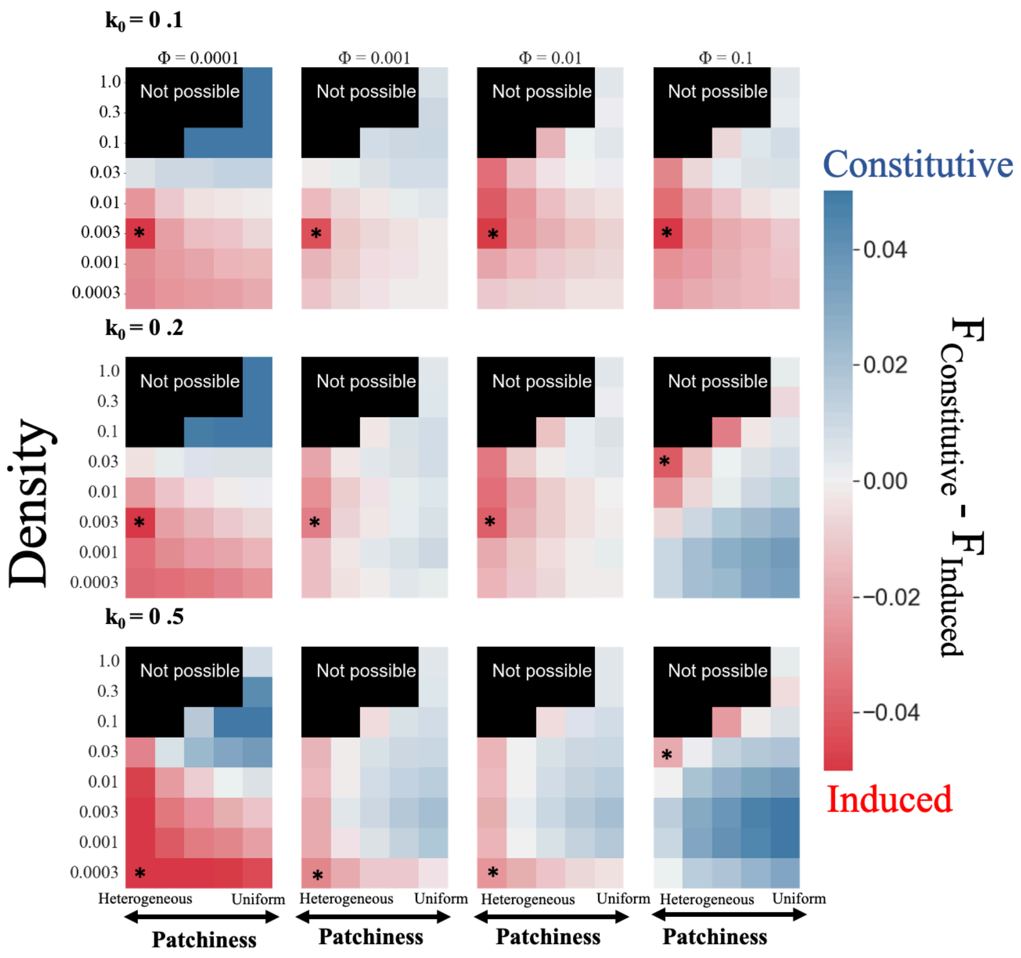

Figure S10. Results for comparison of constitutive and induced defenses, assuming different degradation rates for immune proteins (16-parameter model). The relative fitness of constitutive vs induced defense (color bar) in environments with different patchiness (X-axis) and bacterial density (Y-axis). Induction tends to outperform constitutive defenses when flies encounter bacteria with intermediate frequencies. Induction has a higher fitness in red-colored cells than the best constitutive defense. The highest relative fitness is shown with an asterisk. Heatmaps in the same column show induced responses that were optimized with the same frequency of the sinusoidal input of bacteria (Φ), and heatmaps in each row show the results for the same proliferation rate of bacteria (k_0).

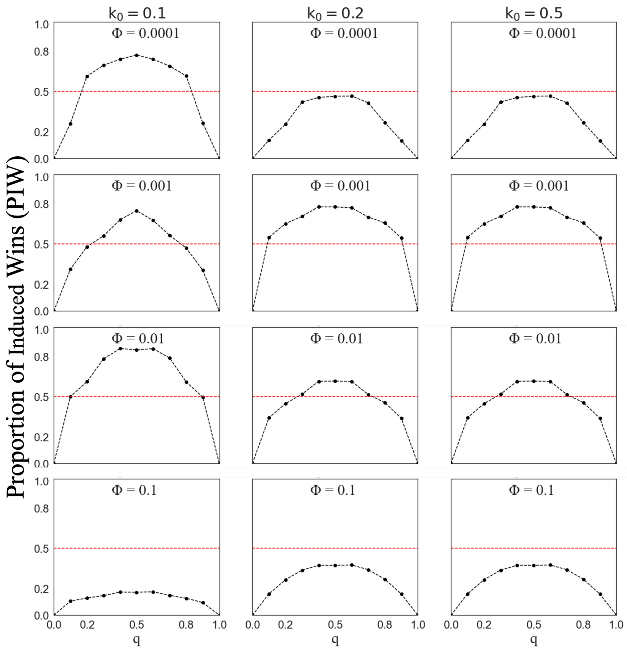

Figure S11. Equal frequencies in two environments favor an induced response. The proportion of times induction outperforms constitutive defense, PIW (Y-axis), is plotted against the probability of inhabiting one of the environments (X-axis). Graphs in a column have the same bacterial proliferation rate (k_0), and graphs in a row have the same Φ value used to to optimize the induced response. Induction outperforms constitutive defense above the dashed line (PIW>0.5), and constitutive defense performs better below the dashed line (PIW<0.5).

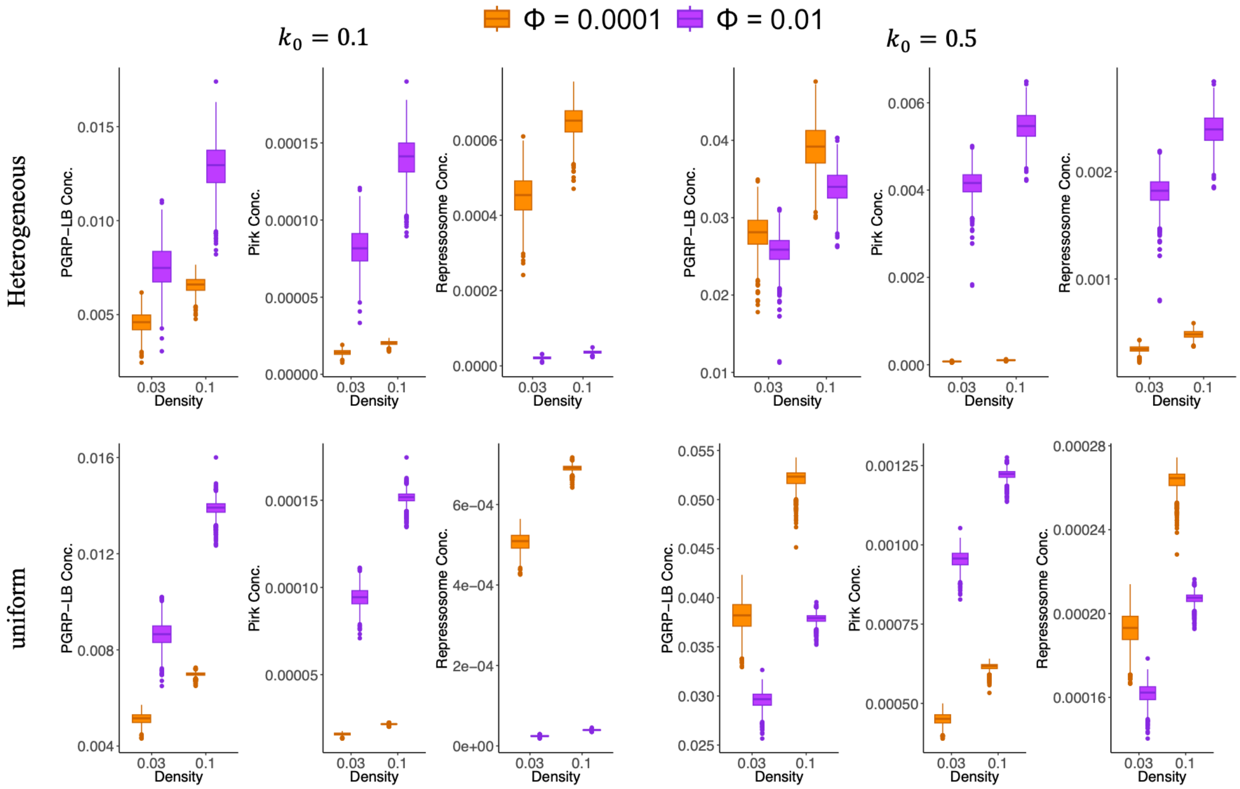

Figure S12. Concentration of proteins involved in negative regulation of the Imd pathway for different optimizations of the induced defense (different values of Φ) across 1,000 simulations for two bacterial densities (0.03 and 0.1). The top row shows the results for the heterogeneous ($p = 1$) distribution of bacteria and the bottom row for the uniform ($p = 3$) distribution of bacteria.

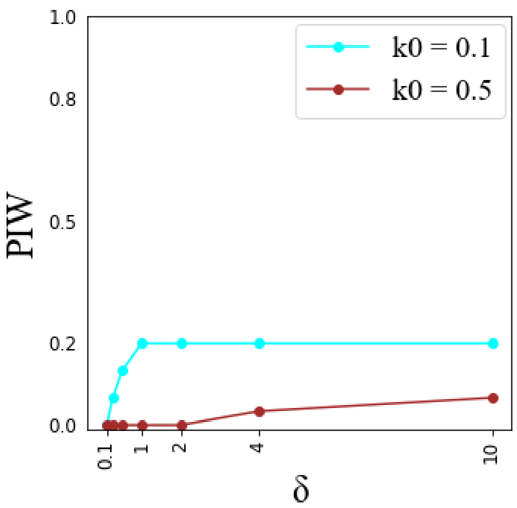

Figure S13. The proportion of induced wins (Y-axis) is plotted against different values of Zs (X-axis). The parameter value is changed by multiplying the optimized values for $\Phi = 0.1$ and $k_0 = 0.5$ by a constant (δ).

Table S1. Optimized parameter values for the 11-parameter model using different frequency of exposure to bacteria (Φ) with different proliferation rates (k_0).

	λ_2	λ_3	R_0	β_1	β_2	β_3	β_4	β_5	β_6	Z_n	Z_s
$\Phi = 0.0001$ $k_0 = 0.1$	2.84752	0.430887	0.000387	3.4e-05	4.941412	0.029356	9.1e-05	0.002898	1.672784	0.000994	0.951317
$\Phi = 0.0001$ $k_0 = 0.2$	2.526267	0.409193	0.000538	3.3e-05	4.874646	0.030043	0.001149	0.000907	1.685706	0.002087	1.035285
$\Phi = 0.0001$ $k_0 = 0.5$	4.951026	2.908026	0.00226	0.000206	4.272663	0.507912	0.001348	0.006272	10.966732	0.000589	5.053662
$\Phi = 0.001$ $k_0 = 0.1$	2.67556	0.403783	0.001202	3.1e-05	0.48815	0.048457	0.001835	0.000932	10.105212	0.006209	1.041554
$\Phi = 0.001$ $k_0 = 0.2$	2.861996	0.713973	0.000485	5.5e-05	4.865809	0.213014	0.01968	0.004068	10.138335	0.010911	0.995953
$\Phi = 0.001$ $k_0 = 0.5$	2.832543	0.748017	0.000526	7.9e-05	4.921888	0.414977	0.020849	0.005438	10.024435	0.008914	4.950373
$\Phi = 0.01$ $k_0 = 0.1$	2.74011	0.783935	0.000102	0.000492	4.962671	0.316262	0.00345	0.000898	10.090715	0.002339	5.001726
$\Phi = 0.01$ $k_0 = 0.2$	2.842505	0.642326	0.00017	0.000253	4.965713	0.352359	0.010237	0.000218	10.093611	0.003855	5.062144
$\Phi = 0.01$ $k_0 = 0.5$	2.839955	0.735402	0.000178	1.8e-05	4.918646	0.427231	0.068726	0.030098	9.93336	0.002599	5.10389
$\Phi = 0.1$ $k_0 = 0.1$	2.457098	0.355084	0.000105	0.000275	5.052434	0.079206	0.004554	0.005398	2.049093	9e-05	1.047421
$\Phi = 0.1$ $k_0 = 0.2$	4.548806	3.389583	0.007199	0.139469	4.049103	1.871999	0.001531	0.003757	11.055369	0.010431	4.923808
$\Phi = 0.1$ $k_0 = 0.5$	4.876191	3.491775	0.000204	0.00148	3.563351	1.770605	0.001946	0.000777	11.039788	0.000147	0.85042

Table S2. The numerical value of density (*d*) and patchiness (*p*) used to simulate bacterial population. Table B contains *d* and *p* values for cells with different cell numbers (cell#), based on table A.

A

Not possible				23
				24
		11	17	25
1	6	12	18	26
2	7	13	19	27
3	8	14	20	28
4	9	15	21	29
5	10	16	22	30

B

Cell #	*p*	*d*
1	1	0.03
2	1	0.01
3	1	0.003
4	1	0.001
5	1	0.0003
6	2	0.03
7	2	0.01
8	2	0.003
9	2	0.001
10	2	0.0003
11	1	0.1
12	3	0.03
13	3	0.01
14	3	0.003
15	3	0.001
16	3	0.0003
17	2	0.1
18	4	0.03
19	4	0.01
20	4	0.003
21	4	0.001
22	4	0.0003
23	1	1.0
24	1	0.3
25	3	0.1
26	5	0.03
27	5	0.01
28	5	0.003
29	5	0.001
30	5	0.0003
